# A behavioral study of live captured coypu (*Myocastor coypus*) and raccoons (*Procyon lotor*) with respect to animal welfare

**DOI:** 10.3389/fvets.2025.1619604

**Published:** 2025-07-30

**Authors:** Franziska M. Schöttes, Friederike Gethöffer, Daniel Tost, Nicole Kemper, Ursula Siebert

**Affiliations:** ^1^Institute for Terrestrial and Aquatic Wildlife Research, University of Veterinary Medicine Hannover, Hannover, Germany; ^2^Institute for Animal Hygiene, Animal Welfare and Farm Animal Behavior, University of Veterinary Medicine Hannover, Hannover, Germany

**Keywords:** live trap, distress, behavior, ethology, capture impact, invasive species, wildlife management, cortisol

## Abstract

Since 2016, coypu (*Myocastor coypus*) and raccoons (*Procyon lotor*) have been listed as invasive alien species (IAS) of European Union concern (The Implementing Regulation (EU) 1141/2016). The three-stage management plan stipulates the prevention of the further spread of species already established in Germany (Regulation (EU) 1143/2014 of the European Parliament and of the Council). Live trapping is a commonly used instrument to catch animals in hunting practice, but also in species conservation and pet protection. As part of a comprehensive study to improve animal welfare in live trapping, this paper focuses on a behavioral study with the aim of assessing the behavior of trapped animals in relation to stress. Video recordings were analyzed using a species adjusted ethogram and a quantitative observation method with focus on the animal in the trap over a maximum six-hour period. Blood and hair samples were taken for endocrinological examinations. The results showed large species-specific and individual differences in the expression of a wide range of behavior and coping strategies. As part of the stress assessment, it was concluded that external factors, among others the type of trap, have an influence on the behavior of coypu and raccoon. The raccoons showed different behaviors depending on the individual data. The endocrinological examinations of the stress parameters cortisol and dehydroepiandrosteron (DHEA) measured in serum and hair revealed differences between the species, indicating differing basal values. For coypu, the measurements indicated differences in serum and hair cortisol levels between juvenile and adult coypu. The study shows substantial indicators, such as the design of the trap type and the duration of capture, can be used to contribute to improve practices in live capture of (wild) animals.

## 1 Introduction

The Coypu (*Myocastor coypus*) and the raccoon (*Procyon lotor*) are established Invasive Alien Species (IAS) in all federal states of Germany (1–3), their population having increased considerably over the last decades (4). Both animals are classified as IAS of European Union concern under European regulation 1143/2014 (2). According to this regulation, an implementation of management following the guidelines of prevention, early detection and eradication is required (5). In Germany, coypu and raccoon are subjected to hunting law in most federal states (6). In Lower Saxony, hunting seasons for coypu and juvenile raccoons are open all year; closed seasons only exist for adult raccoons (7). A recently published survey among hunters from Lower Saxony shows percentages of live trappings ranging from 44–74% (8, 9).

The international standard reflecting animal welfare in trapping is represented by the Agreement of Human Trapping Standards (AIHTS) for the trapping of mammals in restraining and killing traps (10). The AIHTS is controversially discussed, as it does not take into account all welfare aspects nor does it consider the current understanding of animal welfare sufficiently (11, 12). Among others, the AIHTS disregards many physiological indicators (e.g., vital parameters, enzyme and hormone levels) and keeps the behavioral indicators for poor animal welfare to a minimum. So far, research has focused on the practicability and efficiency of trapping (13–16), but increasingly, researchers emphasize the importance of more comprehensive knowledge about welfare aspects for several species, e.g., ethical approaches like the humaneness of methods in trapping (17–21). Animal welfare has gained more attention in public awareness in many countries over the last decades, not only in livestock and laboratories, but also in wildlife conservation and hunting practice (22–25).

Research on animal welfare has been significantly influenced by the popular “model of five freedoms,” which focuses on livestock and its avoidance of negative experiences (26, 27). Its further development went towards a “Five Domains” model, which includes the internal and external conditions and focuses on the associated psychological experiences (28, 29). The updated five domains model includes nutrition, the physical environment, health, and behavioral interactions, which form the basis of the mental state of animals (30). This model also serves as a guideline for improved welfare in zoological facilities and the wildlife conservation sector (31, 32).

Almost one hundred years ago in 1936, Selye started to define and explore the meaning of stress (33, 34), after discovering the relationship between certain pathological findings and the activation of the hypothalamic–pituitary–adrenal-axis (HPA). Even then, he differentiated between positive (eustress) and negative (distress) expressions of stress (35). Ever since, researchers have successfully deduced the physiological factors and signal cascades involved, discovering the complex interplay of nervous, endocrine and immune mechanisms. Within this interplay, the sympathetic-adreno-medullar axis and the hypothalamus-pituitary–adrenal axis are defined (36). So far, the sympathetic nervous system influences numerous organs, generally by mobilizing energy. The transmitters (catecholamines) are responsible for, for example, the increase in the heart rate and blood pressure and a redirection of blood flow (targeting on skeletal muscles and reducing gastrointestinal organs) in order to ensure a quick flight, while pain perception is temporarily decreased. Meanwhile, by the release of glucocorticoids, the metabolism is regulated, suppressing the immune system and generating energy. While the sympathetic nervous system acts within seconds, the rise in, for example, cortisol in the blood occurs within minutes (37). Modern research tries to measure transmitters, changes of immune cell numbers, or mediated chemicals as well as of hormonal blood concentrations (38–41). Behavioral approaches to assess stress in animals are common in the laboratory as well as farm animals (42–44). Due to missing empirical data, behavioral stress evaluation in wild animals is rare (45, 46). Nevertheless, especially in (wild) animals, an evaluation and interpretation of these findings are challenging.

By definition, according to Fraser, “an animal is said to be in a state of stress if it is required to make abnormal or extreme adjustments in its physiology or behavior in order to cope with adverse aspects of its environment and management.” (47). The way of dealing with an unpleasant situation, also referred to as coping, is divided into different approaches: escape, remove, search, wait (48). Coping strategies are not only perceivable in connection with stress-related behavior, but also part of the concept of behavioral adaptation (49). Therefore, coping is seen as the animal’s ability to compensate for a change of situation (50). When a state is reached that exceeds an animal’s coping behavior in response to a stressor, this is referred to as “strain” in stress research. Over the decades, stress research in animals has paid more attention to the various aspects of the meaning of the word stress and the manifestation of stress in physiological and behavioral expressions (33, 47, 51). The question how to quantify stress, the measurability and the classification of stress levels was the focus of earlier research (52), but even then it became clear that there was no single method that could be used in all situations (53). Nowadays, there is a tendency to include several parameters in order to be able to assess the stress factor in many different ways (54–57).

Both from a hunting perspective and for ethical reasons, it is considered important to analyze the behavior of the animals caught, taking into account animal welfare aspects in order to ensure the best possible practice in live capture. To the authors’ knowledge, there are no behavioral studies of live trapped coypu. Only a few behavioral studies exist that describe species-specific behavioral sequences of free ranging or captive coypu from different countries (58–62), most of which describe activity patterns or movement behavior in terms of home range sizes. For raccoons, the behavior during a catch in foothold traps is briefly described in terms of activity (63). Related studies were conducted for different species or trap designs (20, 46, 64–66).

A comprehensive individual animal observation based on a behavior catalog has not yet been compiled for coypu and raccoons. For wild animal species, often only parts of ethograms are created for different study reasons (67). A standardized ethogram for various species, primarily mammals and birds, was already designed in the past to be able to assess visual behavior patterns of wildlife (68), and the idea of using shared ethograms uniformly is still relevant today (69, 70). Standardized behavior recording is also indicated as a way to regularly monitor well-being for captive animals (71).

There are different approaches for the assessment of stress. In the context of laboratory animal research, pain, suffering, distress, or lasting harm are used as indicators and the stress load is classified on this basis and established in an EU Directive (2010/63, Annex VI). Comparatively, a confinement of laboratory animals for less than 24 h is classified as low severity, but inducing escape and avoidance reactions is expected to cause moderate distress. Due to the differences between laboratory and wild animals as well as environmental factors, the applicability of this guideline on live capture in the field is not given, but could serve as a point of reference. Furthermore, scoring sheets, used in behavioral studies for animal experiments to assess stress load assessment (72, 73), are not defined for coypu and raccoons. Open field tests are another widely used experimental strategy for the quantitative study of behavior, e.g., exploratory or avoidance behavior, as they test reactions to a new, pre-defined environment, divided into categories (74–76), but these are also difficult to transfer to fieldwork.

This study considers animal welfare aspects for coypu in three most frequently used live trap types in Germany. Since the capture of non-target species must be considered, the raccoon was chosen as a representative bycatch species in order to illustrate species-specific differences. The study was designed to implement the basic conditions of the AIHTS, including additional parameters. We focus on the behavior and hormonal response to evaluate the stress of live captured coypu and raccoons in two of five restraining trap designs known in the literature (77, 78). As there is no standardized observation procedure for the species examined in our study, we decided to use the generally valid continuous focus method (79), referring to two-level hierarchy in the ethogram, which enabled us, on the one hand, to be certain that no behavior was overlooked and, on the other hand, to record both the behavioral class and the behavioral pattern in detail. The underlying ethogram was developed according to substantial research in zoo and wildlife animals (80). Our hypothesis assumed that the behavior of the captured animals is influenced by external factors and strain-related parameters, but not by the animal’s individual data.

## 2 Materials and methods

### 2.1 Study area and setup

All animals were captured from 2019 to 2022 at different locations in Lower Saxony with permission granted by 33.8-42502-04-19/3190 from the Lower Saxony State Office for Consumer Protection and Food Safety (LAVES). All trapping sites were characterized by access to running or stagnating water and surrounded by varying habitat features such as meadows, reed beds, forests, and cultivated areas. Three different live trap types were tested for animal welfare aspects: first, a wire grid trap (WG trap; common design used in waterboard associations) with a rocker trigger under the food basket at the head end of the trap. This type of trap was set up without cover. Second, the sheet metal trap (SM trap; “Trapper-Neozoen®,” Raiffeisen Warengenossenschaft Osnabrücker Land eG, Melle, Germany), a closed galvanized steel tube that is released by shifting weight beyond the center of the axle. Third, a square wooden box trap (WB trap; Fuchsfalle.de, Horb am Neckar, Germany), with a rocker trigger. Each trap was combined with a self-developed technical system. This comprised a video system, consisting of a 130° wide-angle camera with 940nm lens (KEYESTUDIO Raspberry Pi Camera, Shenzhen KEYES Robot Co. Ltd., Shenzhen, China) and LED ring (INSTAR IR LED Ring, INSTAR Deutschland GmbH, Hünstetten Bechtheim, Germany), connected to a Raspberry Pi 3® computer (Raspberry Pi 3 Model B+, Raspberry Pi Foundation, Cambridge, United Kingdom), to which a temperature sensor and a surf stick (Huawei E3372, Huawei Technologies Deutschland GmbH, Düsseldorf, Germany) were connected. Images of the catch could be retrieved at any time via an internet connection and received by smartphone. A Python script was set up to operate the system. When an animal was caught by triggering the trap mechanism, the camera started continuous recording via a reed contact sensor. The data were stored on the Raspberry Pi 3® computer. Additionally, a trap alarm (TRAPMASTER professional® and TRAPMASTER professional Neo® EPV Electronics GmbH, Lüdenscheid, Germany) trap detector was installed. An audio recorder (Wildlife acoustics songmeter 4®, Wildlife Acoustics, Inc., Maynard, MA, USA) was placed next to the trap to continuously collect audio data. A common camera trap was set up to monitor the surrounding area, referred to as “additional cameras” in the following text. Only vegetarian baits, like fruit and vegetables, were used to attract coypu and raccoons. The traps were set up all year and during the daytime, except for closed hunting seasons.

### 2.2 Trapping and sampling

When the trap closed, each animal’s behavior was video-recorded of 31 coypus and 8 raccoons for up to 6 h. This corresponds to the maximum duration time in the trap permitted by the authorities. After these 6 h the animals were transferred in a capture box with a locking slide and anesthetized with medetomidine hydrochloride (Domitor®, Vetoquinol GmbH, Ismaning, Germany) and ketamine (Ketamine 10%, Ecuphar GmbH, Greifswald, Germany). Blood and hair samples were taken, and vital parameters such as rectal body temperature, oxygen saturation, heart rate, and respiratory rate were monitored during anaesthesia. For euthanasia, T61® (T61®, MSD Animal Health, Intervet Deutschland GmbH, Unterschleißheim, Germany) was administered intracardially after blood sampling, and a general examination was performed according to a standardized protocol. The individual animals were weighed and body measurements were documented. For comparison, five intracardially obtained blood samples per species from animals shot by hunters that had not previously been in a trap were collected immediately after death as a control group. Serum levels of Cortisol and DHEA were determined at the Clinical Endocrinology Laboratory of the Clinic for Cattle, University of Veterinary Medicine Hannover, Foundation, Hannover, Germany (81, 82). For this purpose, all blood samples were prepared using a Z307® universal centrifuge (HERMLE Labortechnik GmbH, Accel 3, 4500 rpm, 12:00 min). Serum samples were stored at −20°C and analyzed with a radioimmunoassay (RIA) for cortisol (Cortisol RIA IM1841, Backman Coulter Inc., Brea, CA, USA) and Dehydroepiandrosteron (DHEA, DHEA RIA IM1138 Backman Coulter Inc.) after validation (83) within 3 months of storage. For the analysis of the hair Cortisol and DHEA levels, hair samples were sent to the Institute for Doping Analysis and Sports Biochemistry Kreischa (IDAS, Kreischa, Germany). The samples were treated according to the internal protocol of IDAS Kreischa dated 12/29/2021, using a deuterated internal standard for the quantification of each analyte, determining hormone levels with gas chromatography and mass spectrometry coupling (Agilent 1290 Infinity-HPLC with Sciex TripleQuad 6500+, e.g., Agilent GC 7890/ 7000 GC-QQQ). For further examination, the carcasses were x-rayed (GIERTH X-Ray international GmbH, Riesa, Germany) and subjected to pathological dissection to detect capture-related injuries, which will be discussed in a follow-up paper.

### 2.3 Data preparation and analysis

The dataset was prepared using Excel (Microsoft Corporation 2016, Microsoft Excel). Based on the clinical examinations and X-ray, the age of the animals was estimated and classified as juvenile (<1year) or adult (>1year) (see Supplementary material), by body measurements, weight, sexual status (mature or immature), and growth plates (presence or absence) in comparison with literature (84–87). We referred time and season according to Central European Time.

**Figure 1:**
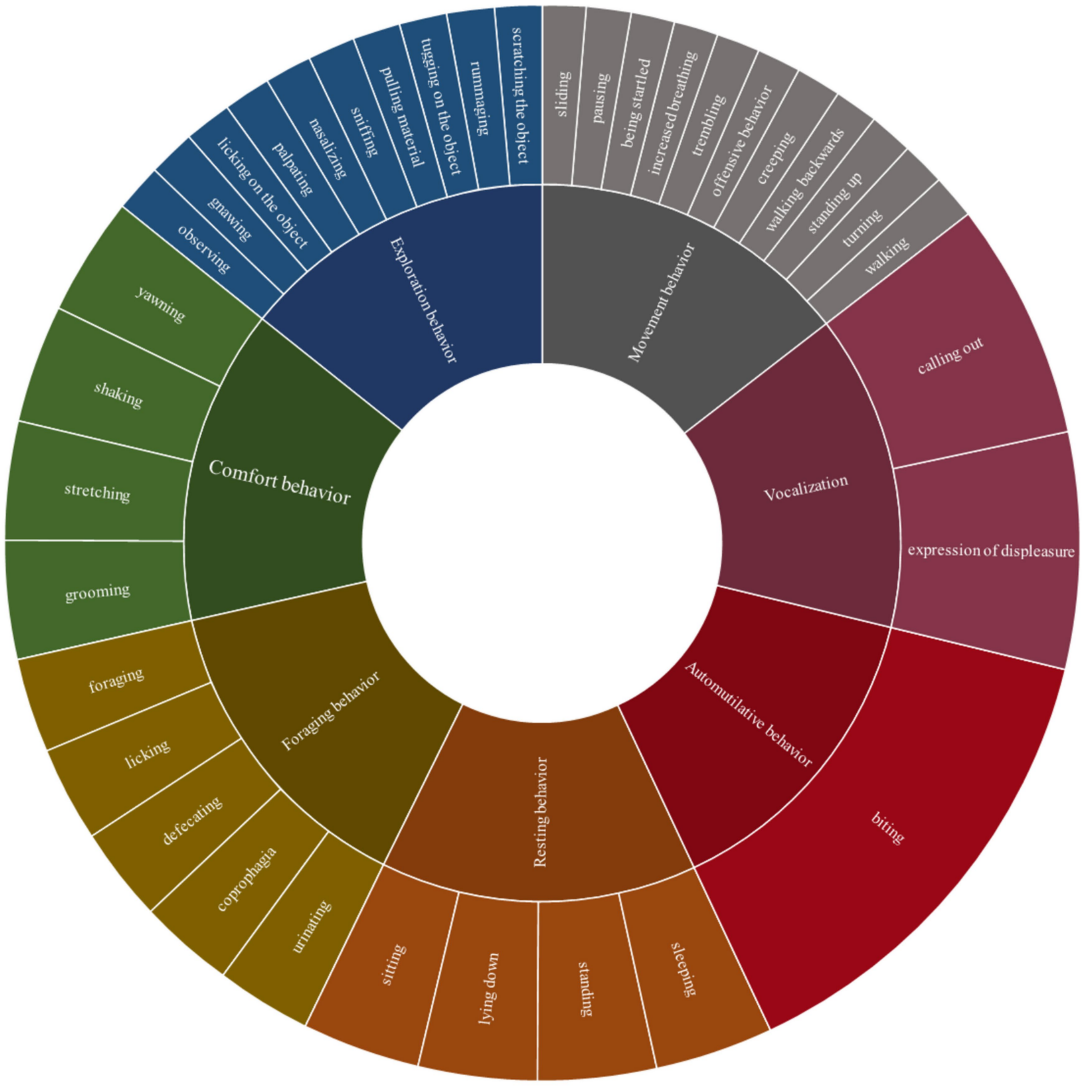
Ethogram with seven behavior classes and associated behavior patterns as basis for video analysis of coypu and raccoon trapped in live traps.

The video recordings were analyzed with the software Mangold INTERACT version 14.3 and 18.7 (Mangold INTERACT, Mangold International GmbH, Arnstorf, Germany). We developed a species-specific ethogram that formed the basis for the behavioral analysis. Thereafter, we categorized the behavior classes into movement behavior, exploratory behavior, foraging behavior, resting behavior, comfort behavior, automutilative behavior, and visualized vocalization in the video (in the following referred to as movement, exploration, foraging, resting, comfort, automutilation, vocalization; see ethogram, Supplementary material Tables S2-5). However, the classes automutilation and vocalization were only included in the descriptive analysis. Each behavior class contained the corresponding individual behavior pattern, such as walking in the movement class, shown in the detailed ethogram (see Figure 1). Individual behavior was defined in terms of frequency and duration, with the start and end times of each behavior pattern documented using a quantitative observation method with focus on the animal in the trap over a maximum six-hour period. The video recordings of six animals caught in pairs in an SM trap were treated separately.

From each class, selected behavior patterns were considered separately. These included nasalizing, gnawing, being startled, trembling, pausing, coprophagia, sleeping, and grooming. These either stood out in relation to stress behavior during the behavioral analysis or had a species-specific reference.

Independent variables were assigned to three groups: individual data, external factors, and strain-related parameters (see Supplementary material Table S1). For the variables weight, rectal body temperature, external temperature, and internal trap temperature, we formed subgroups for better comparability. In our study, there was a data pool of 55 coypu and 48 raccoons, from which only the animals whose capture was recorded in a video were considered in this paper. In order to avoid bias by narrowing down, the entire data pool, including individuals not included in the behavioral analysis, was used as the basis for grouping individual parameters.

In a separate analysis of the acoustic recorder data including all audible vocalizations of the trapped animals, we created a detailed ethogram, and call types of coypu were described for the first time (88). For this reason, the audible vocalizations were not included in the statistical behavioral analysis.

### 2.4 Statistical analysis

To check for observer reliability, we used the Cohen-Kappa (*κ*) test (89), which showed substantial agreement (κ = 0.72) (90).

The behavior classes served as dependent variables for the hypothesis, which was tested in three different models. In these, the individual, external, and strain-related factors were used as independent variables with interactions. Behavioral data were not normally distributed for both species (Shapiro–Wilk >0.05, see Table 1).

**Table 1:** Normal distribution of classes calculated with the Shapiro–Wilk test (>0.05) for coypu and raccoon in duration (d) and frequency (f).

Species	Movement	Exploration	Resting	Comfort	Foraging
Coypu	d: 0.0078 f: 0.0242	d: 0.0506 f: 0.0030	d: 0.0072 f: 0.0989	d: <0.0001 f: <0.0001	d: <0.0001 f: <0.0001
Raccoon	d: 0.0001 f: 0.0061	d: 0.4487 f: 0.5746	d: 0.5434 f: 0.8336	d: 0.1043 f: 0.2107	d: 0.0021 f: 0.0007

SAS Enterprise Guide 7.1 ® (SAS Enterprise Guide, SAS Institute Inc., Cary, NC, USA) (91) was used for all descriptive and R ® (R, R Core Team, Vienna, Austria) (92) for statistical calculations. For statistical modeling of behavioral data, we used the non-parametric simple analysis of variance (ANOVA) and generalized linear models (GLM) with the applied regression data set carData (93). Figures were plotted using ggplot2 (94). The model design was selected based on the good explanation of the effects, the Akaike Information Criterion (AIC), in combination with the Mc Fadden’s R-squared parameter (R^2^), where values >0.60 were accepted. An ANOVA Model was calculated to verify the significance of the variables in the model selection. The level of significance was specified at 0.05. To analyze whether hormonal values of captured animals differed between trap types, we used the non-parametric simple analysis of variance (ANOVA) and Wilcoxon signed-rank test.

## 3 Results

### 3.1 Coypu

#### 3.1.1 Capture data

A total of 31 videos of coypu trapped in the WG trap (n = 17), the SM trap (n = 11), and the WB trap (n = 3) were analyzed. The sex ratio was balanced (16 female/15 male) and the adults in the video (21) outnumbered the juveniles (10). All further recorded individual, external, and strain-related data are listed below (see Tables 2–4).

**Table 2:** Animal-related data of live captured coypu are depicted in this table.

Animal-related data	Total^*^		Dataset (n)
Sex	31		
		Male	16
		Female	15
Age [yy]	31		
		Adult	21
		Juvenile	10
Weight [kg] Weight class	31		
		Light	6
		Medium	13
		Heavy	12

*: *p* < 0.05, else not significant.

**Table 3:** External factors of live captured coypu are depicted in this table.

External factors	Total^*^		Dataset (n)
Temperature outside [°C] Tout	30		
		T0	7
		T1	11
		T2	12
		NA	1
Weather conditions	31		
		Dry	15
		Cloudy	5
		Rainy	6
		Foggy	3
		Windy	2
Season [meteorological]	31		
		Spring	7
		Summer	0
		Fall	12
		Winter	12
Daytime [Central European Time]	31		
		Morning	2
		Midday	1
		Afternoon	4
		Evening	11
		Night	13

*: *p* < 0.05, else not significant.

**Table 4:** Strain-related data of live captured coypu are depicted in this table.

Strain-related parameters	Total^*^		Dataset (n)
Rectal body temperature [C°]Trec	30		
		Low	7
		Medium	10
		High	13
		NA	1
Temperature trap inside [C°] Ttrap	11		
		Tt0	0
		Tt1	5
		Tt2	6
		NA	20
Serum Cortisol [nmol/l] SCortisol	30		
		SC1	12
		SC2	10
		SC3	8
		NA	1
Full body index GS	31		
		GS0	12
		GS1	11
		GS2	7
		GS3	1

* Of individuals considered in behavioral analysis of this paper; NA = Not available.

#### 3.1.2 Behavioral data

In total, 179 h of video footage were recorded, with a mean video length of 5.77 h per catch. Broken down by trap type, the sample size number for the WB trap was very small and therefore assessed to a limited extent.

**Figure 2:**
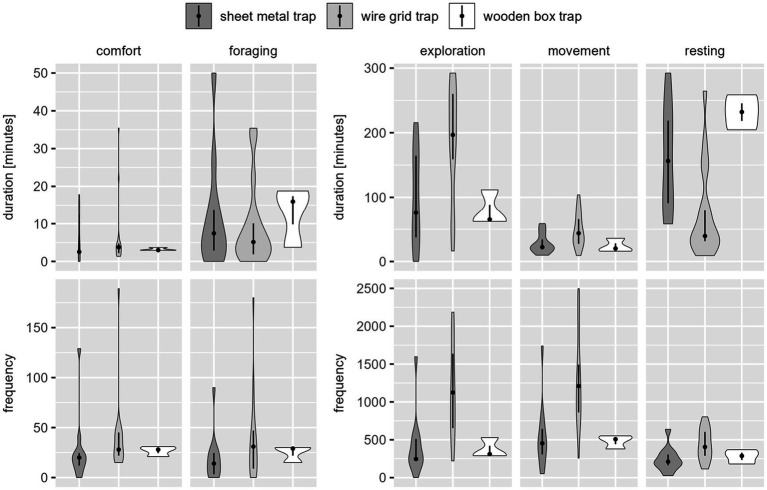
Distribution of observed duration [min.] (above) and frequency [number of events] (below) of the five behavior classes per catch, per trap type for coypu. The dot indicates the median, the line indicates the 25–75% quantile range and the violin shows the distribution of observed durations or frequencies. Movement was expressed in high frequencies with a low mean duration. Exploration was designated with a lower frequency, but lasted longer on average. Resting was shown less frequently, but with long duration times. Comfort and foraging had both a shorter duration and frequency.

There were large differences depending on whether duration or frequency of each behavior class was assessed (see Figure 2).

##### 3.1.2.1 Individual data

For all behavior classes, an influence of the individual factors (age, sex, weight class) was rejected due to AIC and R^2^ value below the limit range of the individual data model. The strain-related variables had no influence on the duration or frequency of the expression of the behavior classes.

##### 3.1.2.2 External factors

**Figure 3:**
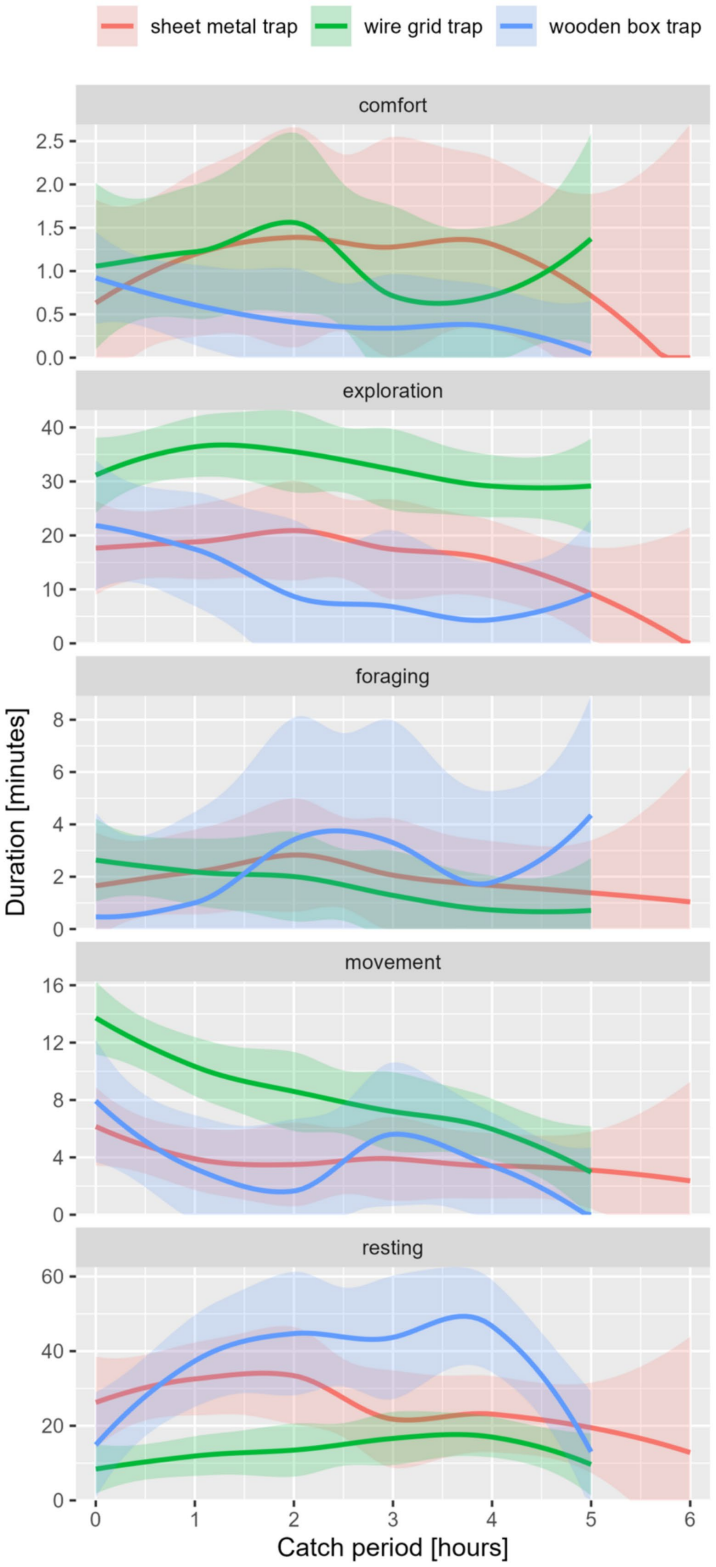
Behavior classes in duration [min.] per catch period [maximum 6 h] of coypu, differentiated by trap type.

The external factors, including the type of trap as one of the most important factors, influenced the expression of the five main behavioral classes in coypu (see Figure 3). The differences were significant for the duration and frequency of exploration and resting behavior and frequency of movement (see Table 5). Significantly high duration of movement was detected in WG or SM traps. A high frequency of movement, exploration, and resting was observed in the WG trap.

**Table 5:** Results of the ANOVA model showing the expression of behavior classes classified by trap type for coypu.

Behavior class	Duration		Significance	Frequency		Significance
	Pr>ChiSq	DF		Pr>ChiSq	DF	
Movement	0.0847	2		< 0.0001	2	***
Exploration	< 0.0001	2	***	< 0.0001	2	***
Comfort	0.5169	2		0.5008	2	
Foraging	0.7085	2		0.3911	2	
Resting	< 0.0001	2	***	0.0008	2	***

***: *p* < 0.001.

Impact of external factors on shown behavior was evaluated using GLMs (see S6-S9). Catches in a WG trap resulted in significantly higher frequencies of movement (*p*-value [f] = 0.002), exploration (*p*-value [f] = 0.000), and resting (*p*-value [f] = 0.007). Low durations of exploration were detected in spring trapping in the WG trap (*p*-value [d] = 0.024). The duration of comfort was lower when trapping in the WG trap in combination with low outside temperatures (*p*-value [d] = 0.037).

The seasons only had a minor influence on the behavioral classes of coypu. The duration of the resting period was shorter when the animals were caught in winter, but not significantly (*p*-value [d] = 0.100). Higher Frequencies in exploration (*p*-value [f] = 0.006) and resting (p-value [f] = 0.014) were recorded during catches in winter.

The influence of daytimes, like trapping at midday (*p*-value [d] = 0.099) or afternoon (*p*-value [d] = 0.002), resulted in an increase in duration in movement. Midday catches also led to an increase in frequency in movement (*p*-value [f] = 0.005), exploration (p-value [f] = 0.049), and resting (*p*-value [f] = 0.005). Catching at night led to a significant increase in duration of exploration (*p*-value [d] = 0.017). Catches during the morning showed a significantly increased frequency of comfort (*p*-value [f] = 0.043). The duration of resting was significantly higher during dry weather (*p*-value [d] = 0.040).

No self-inflicted injuries were detected in coypu. There were 14 vocalizations, which were visible in the video of coypu trapped in the WG trap, with a total duration of 0.86 min, 69 in the SM trap with a total duration of 63.78 min and none in the WB trap.

All animals expressed a wide variety of different behavioral patterns. The values for duration and frequency showed that the patterns were generally expressed very briefly and repeatedly, which indicates continual switching between each.

As expected, exploration was expressed differently depending on the species. As coypu primarily expressed exploration by use of its nose, we named this behavior “nasalizing.” It was expressed very frequently (*p*-value [f] = 0.000) and with significantly high duration (*p*-value [d] = 0.001), particularly in the WG trap. As a more intensive expression of exploration, gnawing was expressed less frequently, but with significantly high duration (*p*-value [d] = 0.017) and frequency (*p*-value [f] = 0.005) in the WG trap and, in contrast, only rarely or not at all in the other trap types. All other behavioral patterns considered here were expressed much less frequently. Although not reaching significance or high numbers, “being startled” was mainly and longest shown by coypu caught in the SM trap, “trembling” was only expressed twice in SM trap.

Seventeen of 31 coypu showed coprophagia in the trap. It was shown in the WB trap with significantly high frequency (*p*-value [f] = 0.012) but occurred in all trap types. Sleeping animals were only discovered in the closed trap types (SM, WB), but in few cases. Grooming was frequently expressed in all traps.

The behavioral patterns “nasalizing,” “gnawing,” and “coprophagia” differed significantly in duration and frequency between trap types (see Table 6 below).

**Table 6:** Differences between the behavioral patterns calculated in one way analysis, classified by trap type for coypu.

Behavioral pattern	Duration		Significance	Frequency		Significance
	*p*-value	DF		*p*-value	DF	
Nasalizing	0.0015	2	**	0.0003	2	***
Gnawing	0.0168	2	*	0.0050	2	**
Coprophagia	0.0110	2	*	0.0121	2	*

***: *p* < 0.001, **: *p* < 0.01, *: *p* < 0.05, else not significant.

#### 3.1.3 Paired catches

On three occasions, two juveniles were jointly trapped in the SM trap. These could be assigned to the same litter and were all caught at the same location during winter. Females predominated, with a ratio of 6:1 (f/m). All individuals belonged to the light weight class. Three showed low rectal body temperatures (32.9°C, 33.0°C, 32.9°C), while three exhibited medium values (34.1°C, 34.4°C, 34.7°C).

**Figure 4:**
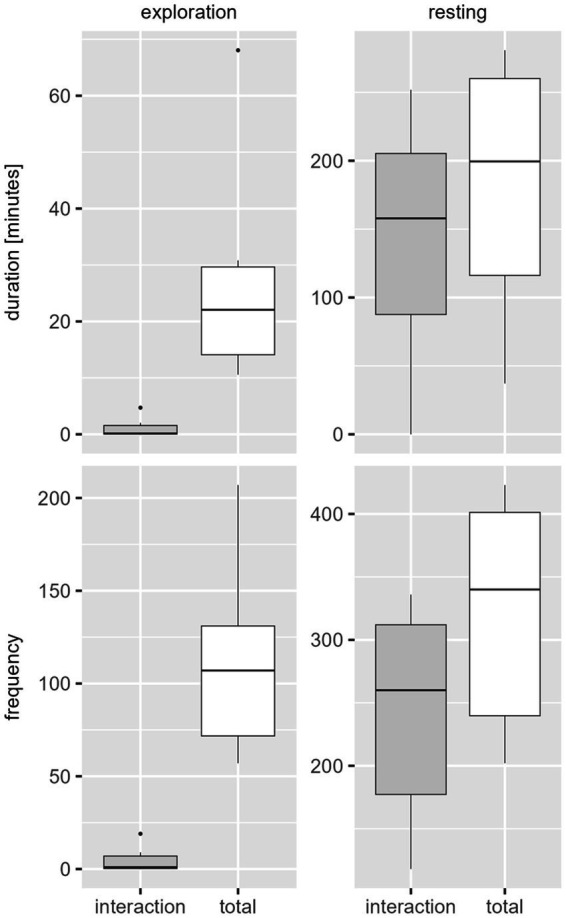
Behavior classes in duration [min.] (above) and frequency [number of events] (below) per catch period of paired trapped juvenile coypu. The white boxplots contain the overall behavior (including the interactive behavior), the gray boxplots contain only the interactive behavior in respect to exploratory and resting behavior.

Movement of all six animals was expressed with little duration (d mean 45.28 min) but high frequency (f mean 441.83). Exploration (d mean 27.73 min; f mean 112.83) and resting (d mean 180.93 min; f mean 322.17) were both detected in high duration and frequency, little comfort (d mean 3.20 min; f mean 14.33) and foraging (d mean 11.25 min; f mean 26.67) was displayed. Among the interactive behavior of the animals caught in pairs, exploration and resting occurred most frequently and long lasting, resting representing the highest proportion (Figure 4).

Apart from interactive behavior in comparison with single catches of juveniles, the animals trapped in pairs showed significantly higher resting and exploration in terms of duration and frequency of behavioral classes. Movement was expressed with longer durations, but less frequently than in single-caught animals. Comfort behavior was shown with less duration and frequency. The foraging behavior was recorded with the same frequency but shorter duration for paired caught coypu.

#### 3.1.4 Occurrence of animals outside the trap

There were 42 sightings of animals, which occurred outside the traps, during 12 catches of coypu. In order to avoid a bias between the open and closed trap types, only observations of animals made by additional camera devices were included.

The average dwelling time of these animals amounted to 7.66 min per presence. In total, the animals were present for an average of 26.83 min per trapping period (maximum 6 h). There was no prominent time of occurrence during the catch (see Table 7). The species recorded included 24 coypu (*Myocastor coypus*), 13 rats (*Rattus norvegicus*), three mallard ducks (*Anas platyrhynchos*), and two songbirds (*Passeri* spp.), which were not determined further.

**Table 7:** Frequencies of sightings of coypu outside the traps, by trap type, and hours of the trapping per catch period.

Interval (hours)	Wire grid trap	Sheet metal trap
0	5	1
1	0	2
2	1	3
3	4	0
4	6	1
5	0	0
6	1	0

Most sightings of coypu were documented during a catch in the WG trap (17/5), fewer in SM trap (7/4), and none during a catch in the WB trap (0/3) (see Supplementary material).

**Figure 5:**
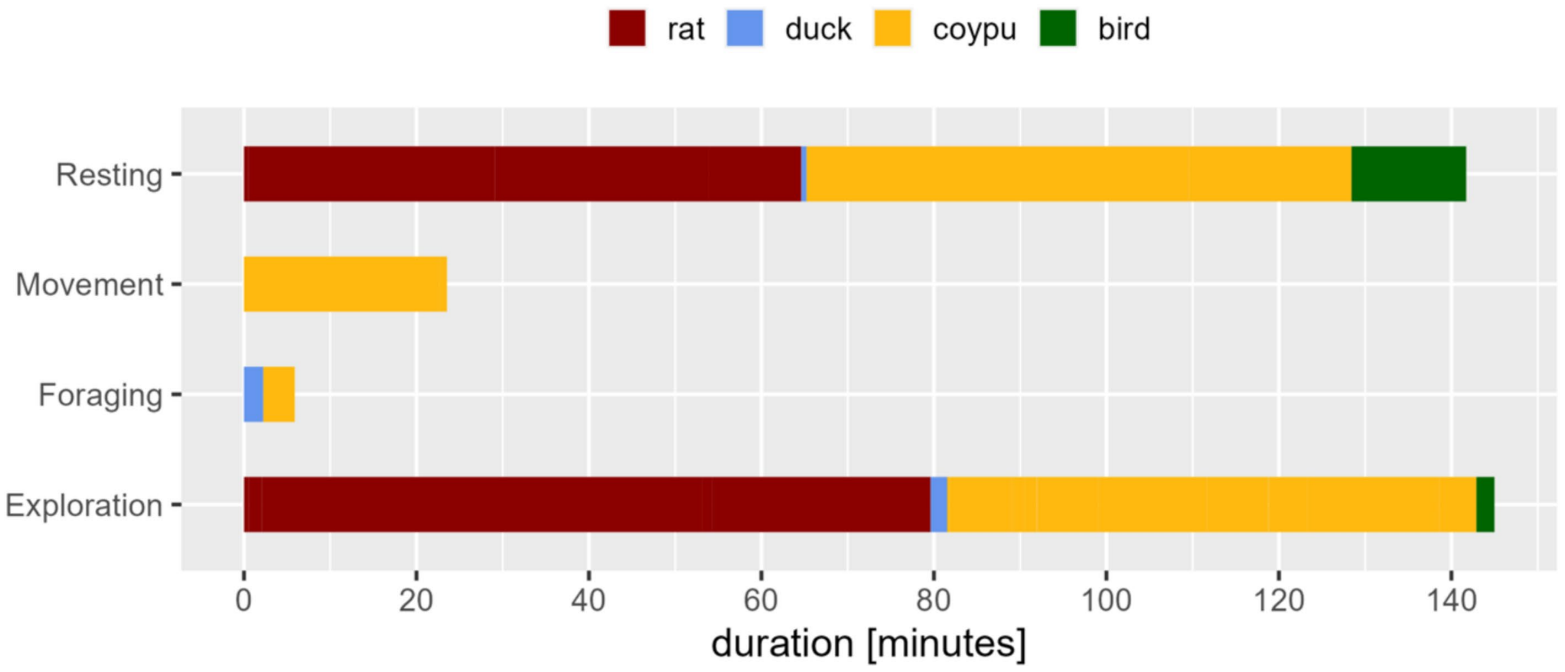
Expressed behavior in classes of the trapped animals during the presence of another animal in the environment, displayed duration in minutes. Especially exploration/movement or resting behavior, in this case sitting, was presented. Comfort behavior was not observed during presence of other animals.

During the animals’ occurrence outside the trap, the trapped animals displayed exploration and resting (see Figure 5). Among these, “observing” and “sitting” were prominently detected.

### 3.2 Raccoon

#### 3.2.1 Capture data

Of eight raccoons that were trapped, five were caught in the WG trap and three in the WB trap. The sex was distributed in favor of males 1/7 (f/m). Of these, seven animals were estimated to be juvenile and one adult (see Supplementary material for individual data).

#### 3.2.2 Behavioral data

**Figure 6:**
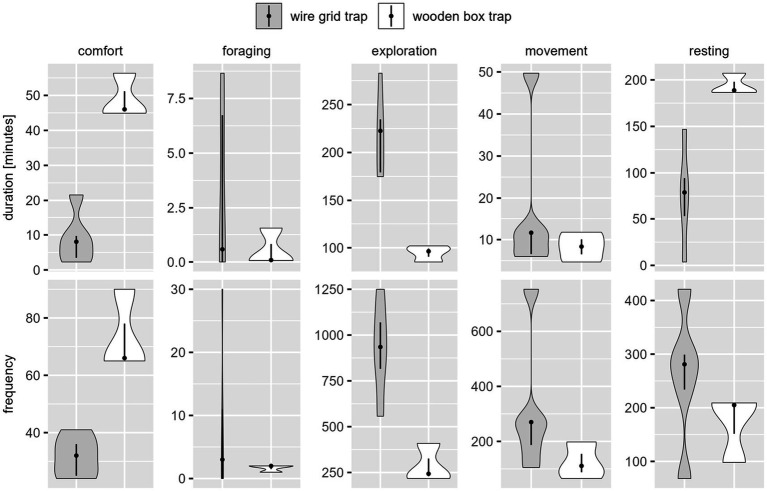
Distribution of observed duration [min.] (above) and frequency [number of events] (below) of the five behavior classes per catch, per trap type for raccoon. The dot indicates the median, the line indicates the 25–75% quantile range, and the violin shows the distribution of observed durations or frequencies. The time duration of exploration was long and of high frequency, followed by resting behavior with a long duration and slightly lower frequency. Movement, comfort, and foraging were shown with a low duration. Movement was detected in high frequency, comfort and foraging with very low occurrence.

A total of 34.71 h of video material was analyzed. Each video lasted 4.34 h on average. Due to the low sample size, these results could be used only to a limited extent for behavior assessment of the raccoons and therefore serve as an approximation of species-specific differences. The differences in the durations and frequencies of the behavioral classes can be seen in Figure 6.

The models indicated that individual data as well as external variables had influence on raccoons behavior in the traps. The model including strain-related influences was rejected for all classes.

##### 3.2.2.1 Individual predictors

The individual data had great influence on the behavior classes for the raccoon. Adult raccoons showed significantly more frequent movement, resting, foraging and comfort. Juveniles expressed foraging significantly longer and frequenter.

Females showed a significantly higher frequency of movement (*p*-value [f] = 0.003), resting (*p*-value [f] = 0.064), and foraging (*p*-value [f] = 0.020). Duration of movement (*p*-value [d] = 8.300) and foraging (*p*-value [f] = 0.008) was also higher in females. In contrast, males displayed significantly shorter duration and less frequency of movement (*p*-value [d] = 7.360; [f] = 0.001) and foraging (*p*-value [d] = 0.004; [f] = 0.012).

Animals of high weight class showed significantly high foraging in terms of duration (*p*-value [d] = 0.008) and frequency (*p*-value [f] = 0.020). Resting (*p*-value [f] = 0.064) and comfort (*p*-value [f] = 0.009) were detected with significantly higher frequency in heavy weight classes. Increased duration of movement could be seen in medium (*p*-value [d] = 0.045) and heavy weight animals (*p*-value [d] = 8.300). In addition, medium weight animals displayed shorter duration times of movement (*p*-value [d] = 0.045). In light (*p*-value [d] = 0.002; [f] = 0.000) or medium weight animals (*p*-value [d] = 0.002; [f] = 0.000), foraging occurred in shorter duration and less frequently.

##### 3.2.2.2 External predictors

**Figure 7:**
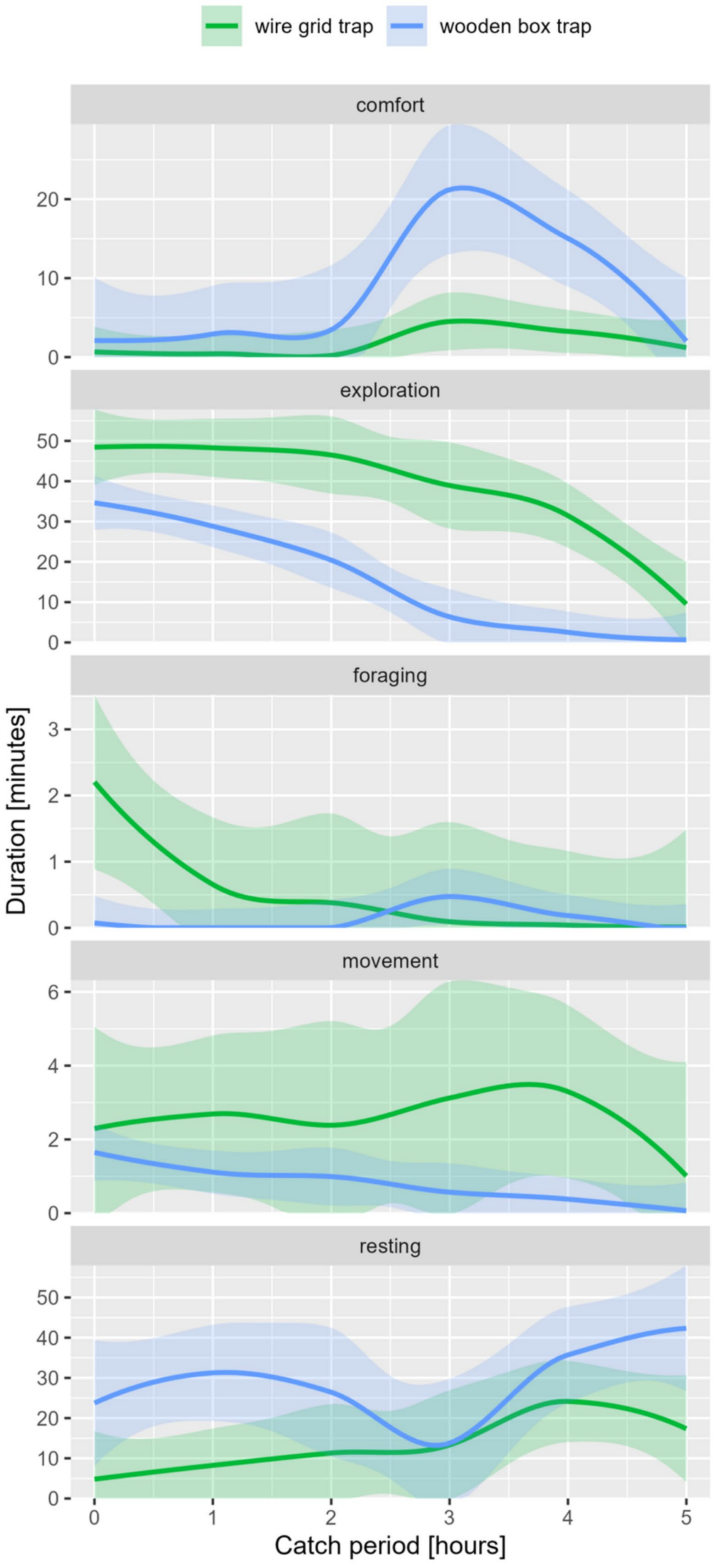
Behavior classes in duration [min.] per catch period [maximum 6 h] of raccoon, differentiated by trap-type.

The trap type was one of the main influencing variables of the external factors. Duration and frequency of exploration, resting, and comfort significantly differed between trap types (see Table 8; Figure 7).

**Table 8:** Results of the ANOVA model showing the expression of behavior classes classified by trap type for raccoon.

Behavior class	Duration		Significance	Frequency		Significance
	Pr>ChiSq	DF		Pr>ChiSq	DF	
Movement	0.5018	1		0.3889	1	
Exploration	< 0.0001	1	***	< 0.0001	1	***
Comfort	< 0.0001	1	***	< 0.0001	1	***
Foraging	0.5211	1		0.1104	1	
Resting	< 0.0001	1	***	0.0001	1	***

***: *p* < 0.001.

Animals caught in the WG trap showed significantly more frequent comfort (*p*-value [f] = 0.000) and resting (*p*-value [f] = 0.010) and higher durations of exploration (*p*-value [d] = 0.040) and foraging (*p*-value [d] = 0.004). In the WB trap, resting (*p*-value [f] = 0.045) and comfort (*p*-value [f] = 0.001) occurred more frequently. In addition, higher duration could be seen in comfort behavior (*p*-value [d] = 0.002).

The trap type was one of the main influencing variables of the external factors. Duration and frequency of exploration, resting, and comfort significantly differed between trap types (see Table 8).

Animals caught in the WG trap showed significantly more frequent comfort (*p*-value [f] = 0.000) and resting (*p*-value [f] = 0.009) and higher durations of exploration (*p*-value [d] = 0.040) and foraging (*p*-value [d] = 0.004). In the WB trap, resting (*p*-value [f] = 0.045) and comfort (*p*-value [f] = 0.001) occurred more frequently. In addition, higher duration could be seen in comfort behavior (*p*-value [d] = 0.002).

Similar to the coypu, the behavioral patterns of the trapped raccoons showed short duration times and high frequencies (see Table 9).

**Table 9:** Extracted behavior patterns of the total data set with minimum, median, and maximum values of duration time and frequency, shown in minutes per catch period (maximum 6 h) for raccoon.

Behavior pattern	Duration per catch period			Frequency per catch period		
	Minimum	Median	Maximum	Minimum	Median	Maximum
Sleeping	0.00	25.90	89.53	0.00	1.50	7.00
Shaking	0.04	0.33	0.85	2.00	13.00	28.00
Grooming	1.75	15.51	54.74	8.00	29.50	53.00
Palpating	5.73	14.85	38.01	35.00	82.50	231.00
Pulling material towards itself	0.00	2.88	31.10	0.00	11.00	57.00
Scratching the object	15.33	63.17	104.88	25.00	219.00	377.00

Exploration was primarily expressed by raccoons by their use of paws to explore the surroundings, which was why “palpating” was detected mostly with significant frequencies in the WG trap (*p*-value [f] = 0.045). More intensive exploration was carried out by “scratching the object.” In the WG trap, the raccoon often drew grass material into the trap. During the catch, the raccoons “groomed” themselves significantly more frequently and for longer times in the WB trap (*p*-value [d] = 0.000; [f] = 0.005). “Shaking” was also one of the behaviors shown, but with less duration and frequency, while “sleeping” was shown with high duration, but only very rarely. In the comparison of traps, significantly higher values for the duration and frequency of “sleeping” were detected in the WB trap (*p*-value [d] = 0.039; [f] = 0.022).

The behavior “sleeping” and “grooming” differed significantly in duration and frequency between trap types. “Palpating” showed significant differences in frequency only (see Table 10 below).

**Table 10:** Differences between the behavioral patterns calculated in one-way analysis, classified by trap type for raccoon.

Behavioral pattern	Duration		Significance	Frequency		Significance
	*p*-value	DF		*p*-value	DF	
Sleeping	0.0389	1	*	0.0219	1	*
Shaking	0.1319	1		0.2937	1	
Grooming	0.0003	1	***	0.0053	1	**
Palpating	0.1393	1		0.0447	1	*
Scratching the object	0.0986	1		0.0699	1	

***: *p* < 0.001, **: *p* < 0.01, *: *p* < 0.05, else not significant.

### 3.3 Endocrinological data

#### 3.3.1 Differences between species

**Figure 8:**
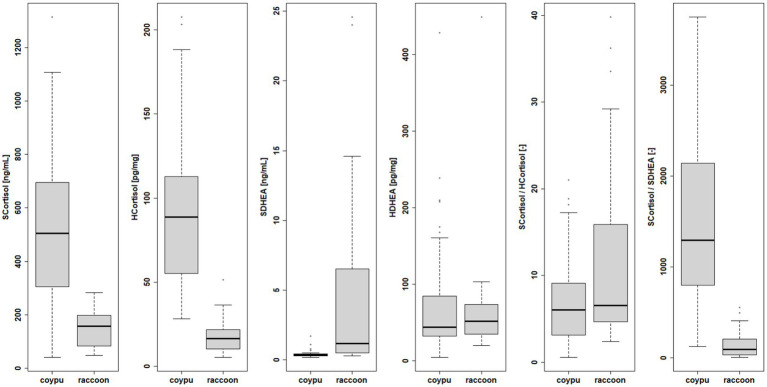
Species differences in endocrine examination of serum and hair cortisol, serum and hair DHEA and quotients.

Endocrine examinations of cortisol and DHEA for 24 raccoons (12 males, 12 females) and 58 coypus (27 males, 31 females) revealed distinct higher levels of serum and hair cortisol and lower serum DHEA levels in coypus than in raccoons (see Figure 8), whereas for hair DHEA, no significant difference could be detected. The serum DHEA concentrations negatively correlated with the cortisol values (see Supplementary material). The serum/hair DHEA quotient was also not comparable between the species for coypus and raccoons, respectively (Wilcoxon *p*-value = 4.341e-05, see Supplements).

#### 3.3.2 Differences within species

**Figure 9:**
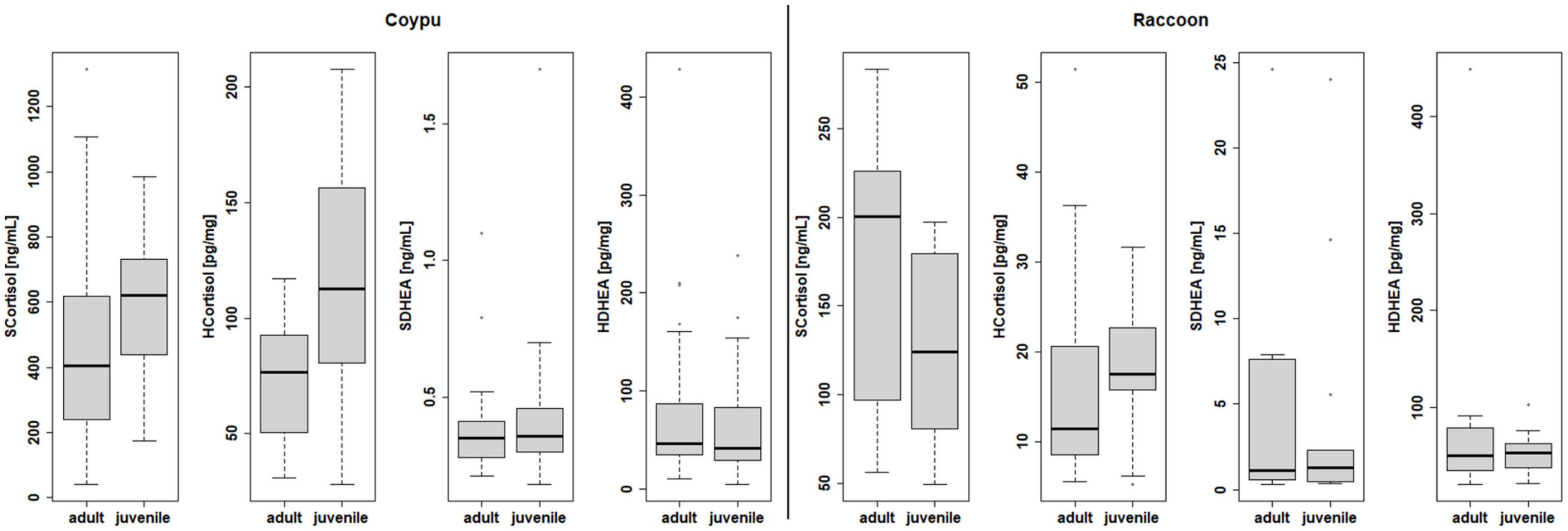
Differences within species in endocrine examination of serum and hair cortisol, serum, and hair DHEA in terms of age.

In order to assess a possible stress reaction, concentrations of cortisol and the steroid hormone DHEA were measured in the serum and in the hair of the examined coypu and raccoons (see Figure 9). The differences in stress parameters measured in serum and hair found between sexes were not statistically significant within the species (see Supplementary material), but age (differentiated between juvenile and adult) did bring significant differences for serum cortisol levels (Wilcoxon *p* = 0.082 raccoons, 0.0369 coypu) as well as for hair cortisol levels (Wilcoxon *p* = 0.0004929) in coypu.

Differences in juvenile (n = 26 blood samples, 27 hair samples) vs. adult (30 blood samples, 31 hair samples) serum and hair cortisol levels were statistically significant in coypu (Wilcoxon *p* = 0.03697; 0.0004929). Differences in juvenile (n = 13 blood samples, 9 hair samples) vs. adult (n = 10 blood samples, 7 hair samples) raccoons serum and hair cortisol levels were not statistically significant (Wilcoxon *p* = 0.1475, 0.351).

There were no differences in hormonal findings between the tested trapped animals and the control group either for coypu or raccoons (see Supplementary material).

## 4 Discussion

As a part of a holistic assessment of animal welfare in trapped coypu and raccoons, this study focused on behavior of these animals during their capture. This paper evaluated the performance of the trapped animals in combination with hormonal and somatically findings, aiming to determine the best practice for stress evaluation. The present study comprises captures of 31 coypu and eight raccoons, showing that both coypu and raccoons exhibit diverse, species-specific, and individual behaviors during capture, which provide evidence of different coping strategies and stress reactions. None of the AIHTS indicators of poor animal welfare occurred consistently, but automutilative and frustration-driven behavior patterns were occasionally observed in raccoons. The combination of behavioral analysis and physiological parameters highlights the need for a differentiated and expanded assessment of stress and animal welfare during live capture.

In our study, we opted for a neutral observational recording of behavior, which ensures a high degree of comparability, by recording behaviors based on a predefined definition, without any interpretation by the observer. However, it is a longstanding realization that the same behavioral expressions can be motivated differently (95). Therefore, the assessment of behavioral patterns may lead to varying main classifications. For example, according to the ethogram, the behavior of “sitting” was categorized into the class of resting behavior, although it should not be used synonymously with resting, but could also express “waiting” or “persevering.” In this way, the allocated meaning of various recorded behaviors can be discussed, especially in relation to stress behavior. We can state that the captured animals expressed all main classes of expected behavior according to the underlying ethogram. For coypu, the categories were comparable to those described in other studies (60, 96). According to the AIHTS on which our study is based, two indicators that serve as signs of poor welfare are stipulated in the assessment of trapped mammals (10). The first indicator, excessive immobility and lack of reaction, was not expressed by coypu or raccoons in this study.

Meanwhile, we found species-specific differences in the behavioral recordings that might hint at coping strategies for both species. Raccoons primarily showed palpating and scratching at trap parts, whereas coypu merely displayed nasalizing and gnawing. In behavioral research, animals` reactions to an aversive situation are classified within the coping concept (97). Recurring striking individual differences in coping style were described for various species as proactive or reactive tendencies with corresponding characteristics (98). Efforts have been made in mouse models (*Mus musculus domesticus*) to genetically breed less aggressive animals or more stress-tolerant animals, which has provided further insights into the link between genes and behavior (99). Nowadays, it is assumed that animals have personalities with differences in behavioral expression between individuals of a species, which are shaped by their genetics, environment, and experiences (100–102). Among all our data, clear individual differences in behavioral patterns were detected. Against the background of individual personalities with individual coping behavior, a stress assessment must therefore be viewed in a more differentiated light.

As expected, species-specific differences were found, particularly with regard to exploration behavior. The raccoons showed behavior already known from other studies (45), in which it in the first place explored with use of its paws and climbed nimbly through the traps. Its mouth and teeth, especially canines, were also used in the processing of the trap materials. On the contrary, the coypu primarily used its nose for exploration, which is why the term nasalizing was added to the ethogram. Its paws were used to explore the trap walls or to dig. Animals that perform a greater number of different motor actions are more successful at solving a new task (103). Raccoons are known for their ability to solve innovative problems, developing multiple solutions to a novel problem (45, 104). This and the ability to alter its behavior in response to environmental stimuli are characteristics of a behaviorally flexible species, which appears to have an influential role on the type and manifestation of coping behavior (105).

In this study, frequency and duration were used to evaluate quantitative and qualitative dimensions of behavior. Remarkably, in most cases, a short duration and high frequency of individual behavior results in a pattern of constantly changing behaviors (20). If the coping strategy of the animal fails, an increase in duration, frequency, and intensity of coping behavior patterns might occur (48). It is also described that animals stuck in a certain situation will try out a variety of coping strategies (48), which in this evaluation becomes particularly evident in the frequent occurrence of exploratory behavior in raccoons and coypu. The short duration and frequent changes of behavioral patterns suggest that an individual behavior is started, but not completed. In addition, it was observed that a behavior that had just been started was interrupted, followed by another behavior that could not be assigned to a trigger and often seemed out of context. This behavior is described in ethology as a displacement activity (106–108), arising from nervousness or a stressful situation and was already described for other animal species (109). A further expression of coping as a response to an aversive situation or due to the influence of external stressors is the expression of abnormal behavior (48). One form, the behavioral stereotypies, are also considered successful coping behavior and are associated with reduced stress (50) although they have not yet been interpreted consistently. Stereotypies could not be observed in this study, but it should be kept in mind that the trapping period was limited.

The second indicator that serves as a sign of poor welfare in the context of AIHTS is automutilative behavior, which is defined as self-directed biting leading to severe injury (self-mutilation). We identified automutilation in raccoons, which manifested as biting or scratching, although occurring in very low numbers. It is apparent that this was done out of frustration at not being able to escape the trap. Additionally, aggressive behaviors that occurred in raccoons, such as tugging at the object, indicate frustration (110–112) and were also considered as an expression of coping strategy (48). Similar behavior, like bar biting or digging, were also documented for other captured species in the literature (64). For coypu, no aggression or automutilative behavior was detected.

It was found that the individual neurophysiological reactions of animals to a stressful situation are also reflected in the activity level of the animals (74, 98). This finding is associated with behavioral differences in a variety of contexts (113). Different levels of “active” or “passive” behavior were observed over the entire trapping period. A distinct trend towards temporal divisions could only be derived descriptively for movement behavior, which was recorded more at the beginning than at the end of the six-hour trapping period. A difference in movement behavior, which may indicate an activity level, could be detected in the case of raccoons. A generally higher level of activity was detected in female raccoons, particularly in frequency and duration of movement compared with male raccoons that showed lower frequencies and duration times of movement behavior. The particularly high activity and resilience in the first moments after trap closure described in other studies could only be observed in few cases (64, 114). Nevertheless, over the course of the maximum six-hour catch we could not confirm any fatigue of the animals with a decrease in exploration activity over a longer trapping period, as described in the literature (63). Also, other behavioral signs that would have indicated habituation to the trap were not observed, so that the assumption that habituation does not take place in such situations can be emphasized (20). It is also assumed that influencing the amount of time the animal is exposed to the stressor has no effect on the measured behavior (115).

In addition to the direct influencing factors, there are many external factors to which the trapped animal is indirectly exposed and which could influence the animal’s behavior. Due to its construction, environmental stimuli (climatic factors like precipitation, temperature, or sunlight as well as acoustic or visual attractions) are best perceivable for trapped animals in the WG trap. Correspondingly, we detected high values especially for movement and exploration. In contrast, the SM trap was isolated from its surroundings, and high resting and comfort was expressed by raccoons in the WB and WG trap. For raccoons, the duration of comfort was higher when caught in the WB trap, supporting the use of a closed trap type. In addition, the wood seemed to have a slightly (sound) insulating effect, which could be beneficial. Evidently, the special feature of the SM trap, a movable pipe, startled the animals when the trap tipped over.

Seasonal variations appeared in frequency of exploratory and resting behavior in coypu and comfort behavior in raccoons. The open trap type exposes the trapped animals to the weather. Coypu caught in the WG trap in windy weather showed higher exploration, slightly missing the significance. They also displayed lower durations of comfort and resting during low outside temperatures or in winter catches. However, a low temperature is associated with a reduced activity phase (60). In contrast, a high movement behavior of the coypu was recorded at low outside temperatures, which could be justified with lack of isolation and thermoregulation.

The daily activity of non-native species may differ in new environments (116), which is why the comparability of extra-European studies are not always given. The coypu and raccoons are considered to be crepuscular and nocturnal species (58, 60, 61, 117, 118). Contrary to what we expected, a diurnal rhythm in form of crepuscular and nocturnal activity of coypu could not be observed in the trapped animals. The duration of movement behavior of coypu was higher when caught at noon or in the afternoon. Longer movement is usually only documented during periods of cold weather, which we could not confirm (119). Longer-lasting exploratory behavior at night in coypu could be associated with physiologically increased activity in the daytime (60). During midday, the activity of coypu increased in frequency of movement and exploration behavior, while comfort behavior showed higher frequencies in the morning. Although trapped raccoons showed more persistent exploratory behavior and foraging in the evening, resting frequencies were also significantly higher, while at night lower resting times were documented, which corresponds to the nocturnal rhythm and the resting times of animals in the wild (120). Interestingly, we found that trapped coypu switched to either exploration or resting behavior if animals outside the trap were present. Here, mostly sitting was encoded (see Supplementary material), which might be interpreted as pausing and vigilance. Sitting is usually expressed in the active period of day (60), which is why an assignment to the behavior class resting can be discussed. Additionally, observing was recorded almost exclusively if other animals were present.

However, the expression of physiological behavior of the targeted species is an important indicator for animal welfare (121). Few behaviors, such as coprophagia (122, 123) could be interpreted as physiological for coypu. Coprophagia in captive coypu is usually conducted daily after returning to the nest in the morning hours or at midday (60), but was rarely expressed during live capture. Sleeping and lying are reported very rarely, probably because these behaviors are mainly expressed in the nest (60). Additional behaviors of social rodents, such as grooming each other (84), cannot be practiced in a single capture. Without doubts, the expression of physiological behavior is limited during the trapping period of 6 h, due to temporally and spatial limitation. The diurnal rhythm cannot be exhibited, and resources like water and nesting material are not accessible. This could also be a reason for the low frequency of encoded grooming, as the animals usually clean themselves after swimming or in the nest (60).

Since both coypu and raccoons are species living in social groups, isolation during live trapping restricts their social behavior. During captures of juvenile coypu pairs, interactive behavior was expressed repeatedly over the capture period. The assumption that two animals might calm each other down could be confirmed, as more resting behavior was shown compared to individually captured juveniles. It also was remarkable that less frequent movement behavior was shown in the paired captures. In addition, social behavior could be observed, mainly expressed by resting behavior as contact lying (60). In the five domains model, the expression of physiological behavior is one of three basic pillars of mental status (121, 124).

Changes in physiological parameters can provide a further measurability of stress. In addition to increased heart rates and body temperatures, an increase in the traditional stress hormone cortisol in serum samples is often described in trapped animals (12, 64, 114, 125). Other biomarkers like DHEA are being established as a method in the latest research, as the interaction of many measuring points allows more complete conclusions to be drawn about the neurological stress reactions (126). In this study, cortisol and DHEA parameters were measured in hair and blood that provide information about both acute and chronic stress reactions. Our results showed no significant differences between the different trap types or sex, although there were significant age-specific differences. However, significantly different serum cortisol and DHEA levels were measured between the two species, indicating different basal levels. The correlations between behavioral expression and neurophysiological reaction described in the literature (98, 99) could not be reflected in our results.

Taking into account European and national legislation, such as the AIHTS, we believe that live trapping does not do sufficient justice to animal welfare. In this study, essential points of reference in the behavior of the animals during live trapping were shown and the complexity of the different parameters are clarified, which is why an all-encompassing consideration of all methods is always necessary. Extended guidelines adapted to the modern understanding of animal welfare are necessary in order to be able to carry out responsible live capture in the future, especially in the area of conflict with invasive species.

## 5 Limitations of the study

This study is limited due to restrictions of the animal experiment to a six-hour trapping time, although a realistic trapping time in practice may last considerably longer. The predefined time limitation in the experimental setup resulted in significantly higher animal welfare standards than those commonly applied in practice, creating a gap that is ethically questionable and must be taken into account when transferring the study’s findings to real-world applications. It was neither possible to carry out uniform behavioral tests nor to use a comparable ethogram nor to generate a large sample size due to field study conditions. A comparison study to access “normal behavior” would be required especially for raccoons, where behavioral studies are rare. The results of this study cannot be generalized, as there are too many differences between the trap types and species, which is why an assessment of all conditions is essential. For these reasons, an assessment of behavior in relation to animal welfare is only possible to a limited extent, which should be taken into account in future studies. Our findings are intended as indications of an improved live trapping of coypu and bycatch and does not fulfill the claim to general validity.

## 6 Conclusion

We conclude that both species-specific and individual behavior patterns, as suggested in this study, can serve as indicators of animal welfare. The design, including insulation, and placement of the trap as well as the handling of the trapped animals also contribute significantly to animal welfare. Therefore, adapting live traps to the specific needs, behaviors, and physiological characteristics of different animal species is essential to ensure animal welfare. Species-specific activity patterns, stress responses and coping strategies should be considered. Only by aligning trapping conditions with the physiological requirements of the animals, stress, injury, or suffering during capture can be minimized. It has been confirmed that trapping (related) species as pairs can improve animal welfare, and therefore it is suggested to support further research on multi-species trapping. We emphasize the consideration of the length of time the animals remain in the trap as relevant to regulation and recommend short intervals. Every practitioner of live trapping is responsible for conscientious and animal-friendly work, which is why we appeal to those who use live traps to consider this during handling.

## Data Availability

The original contributions presented in the study are included in the article/Supplementary material, further inquiries can be directed to the corresponding author.
