# Supplementary material for: A behavioral study of live captured coypu (Myocastor coypus) and raccoons (Procyon lotor) with respect to animal welfare
Source: Front Vet Sci. 2025 Jul 30;12:1619604. doi: 10.3389/fvets.2025.1619604 (PMC12343265; doi:10.3389/fvets.2025.1619604)
Supplement: Supplementary file 1 [file Table_1.docx]

Supplementary Material

# Supplementary Data

## Predictors

Table S1: This table defines the predictors used in the GLM calculation for coypu and raccoon, divided into three models: individual data, external factors, and strain-related parameters. All variables are of categorical type.

| Model | Predictor | Variable | Description |
| --- | --- | --- | --- |
| Individual data | gender | male female |  |
|  | age | juvenile adult | < one year > one year |
|  | weight class | light  medium  heavy | coypu: 0.8-2.0 kg raccoon: 1.5-3.52 kg coypu: 2.1-4.8 kg raccoon: 3.52-6.0 kg coypu: 4.9-6.8 kg raccoon: 6.0-8.4 kg |
| External factors | trap type | wire grid (WG trap) sheet metal (SM trap) wooden box (WB trap) | open trap type closed trap type closed trap type |
|  | season | spring summer fall winter | 03.01.-05.31 06.01.-08.31 09.01.-11.30 12.01-02.29 |
|  | daytime | morning  morning  midday  afternoon  evening  night | ST: 07:00:00-10:59:00/WT: -01h ST: 11:00:00-12:59:00/WT: -01h ST: 13:00:00-14:59:00/WT: -01h ST: 15:00:00-17:59:00/WT: -01h ST: 18:00:00-21:59:00/WT: -01h ST: 22:00:00-06:59:00/WT: -01h |
|  | weather conditions | dry foggy rainy cloudy sunny |  |
|  | temperature outside (Tout) | T0 T1 T2 | -1.0-3.0 °C 3.1-7.0 °C 7.1-18.6 °C |
| Strain-related parameters | rectal body temperature (Trec) | low  medium  high | coypu: 32.1-33.5 °C raccoon: 36.5-38.17 °C coypu: 33.6-35.0 °C raccoon: 38.17-38.9 °C coypu: 35.1-38.81 °C raccoon: 38.9-41.1 °C |
|  | temperature trap inside (Ttrap) | Tt0 Tt1 Tt2 | -0.38-4.4 °C 4.5-8.4 °C 8.5-24.0 °C |
|  | serum cortisol (SCortisol) [nmol/L] | SC1 SC2 SC3 | 40.80- 395.71 395.72-621.67 621.68-1086.96 |
|  | full body index | GS0 GS1 GS2 GS3 | no injuries minor injuries moderate injuries severe injuries |

## Coypu

### Behavioral data

Table S2: Behavior classes for each individual (coypu) in duration and frequency per catch period.

|  | Duration [minutes] | | | | | Frequency [number of events] | | | | |
| --- | --- | --- | --- | --- | --- | --- | --- | --- | --- | --- |
| Animal ID | exploration | resting | comfort | foraging | movement | exploration | resting | comfort | foraging | movement |
| Nu_01 | 158.99 | 79.32 | 1.69 | 12.84 | 103.93 | 805 | 169 | 22 | 47 | 1209 |
| Nu_02 | 164.06 | 156. 33 | 2.29 | 26.94 | 11.93 | 191 | 159 | 12 | 25 | 163 |
| Nu_03 | 75.63 | 84.85 | 2.85 | 4.67 | 49.72 | 551 | 168 | 23 | 14 | 818 |
| Nu_04 | 292.32 | 15.15 | 2.2 | 6.8 | 45.12 | 2185 | 363 | 24 | 50 | 1230 |
| Nu_05 | 164.78 | 71.25 | 17.82 | 49.99 | 5875 | 1597 | 638 | 129 | 90 | 1738 |
| Nu_06 | 225.41 | 37.41 | 4.14 | 35.39 | 60.54 | 2035 | 723 | 23 | 180 | 1835 |
| Nu_07 | 0.12 | 292.35 | 0 | 0 | 27.61 | 1 | 26 | 0 | 0 | 52 |
| Nu_08 | 186.75 | 68.98 | 1.47 | 1.91 | 87.94 | 1976 | 803 | 18 | 8 | 2496 |
| Nu_09 | 65.25 | 222.2 | 2.53 | 2.76 | 19.3 | 467 | 320 | 16 | 3 | 584 |
| Nu_10 | 23.24 | 97.37 | 0.52 | 7.93 | 40.38 | 214 | 84 | 5 | 18 | 453 |
| Nu_11 | 162.59 | 37.03 | 1.69 | 0 | 65.67 | 1317 | 512 | 18 | 0 | 2139 |
| Nu_12 | 34.89 | 260.26 | 1.76 | 15.13 | 19.79 | 239 | 211 | 12 | 24 | 457 |
| Nu_13 | 196.62 | 39.49 | 2.95 | 4.04 | 38.25 | 654 | 210 | 25 | 9 | 742 |
| Nu_14 | 284.76 | 45.42 | 2.51 | 0.19 | 27.46 | 848 | 249 | 16 | 1 | 880 |
| Nu_15 | 268.33 | 33.85 | 4.18 | 5.14 | 43.78 | 1124 | 473 | 46 | 33 | 1412 |
| Nu_16 | 178.74 | 146.27 | 3.97 | 4.34 | 27.2 | 900 | 289 | 45 | 27 | 1109 |
| Nu_17 | 276.15 | 31.55 | 6.59 | 1.18 | 45.58 | 1636 | 657 | 41 | 9 | 1328 |
| Nu_18 | 210.15 | 18.06 | 3.77 | 8.64 | 81.25 | 1852 | 666 | 47 | 31 | 2310 |
| Nu_19 | 260.04 | 79.84 | 22.12 | 5.82 | 20.69 | 1260 | 604 | 52 | 12 | 698 |
| Nu_20 | 182.44 | 58.27 | 2.28 | 0.05 | 19.47 | 357 | 214 | 21 | 1 | 380 |
| Nu_21 | 62.15 | 258.74 | 3.68 | 15.96 | 15.89 | 289 | 180 | 31 | 30 | 379 |
| Nu_22 | 65.15 | 232.1 | 3 | 18.78 | 35.95 | 311 | 287 | 21 | 29 | 508 |
| Nu_23 | 111.61 | 204.6 | 2.93 | 3.73 | 20.08 | 529 | 369 | 28 | 15 | 552 |
| Nu_24 | 215.63 | 108.54 | 12.1 | 3.02 | 22.23 | 561 | 359 | 26 | 4 | 700 |
| Nu_25 | 127.55 | 197.97 | 12.87 | 12.39 | 9.74 | 220 | 190 | 40 | 28 | 234 |
| Nu_26 | 258.11 | 9.13 | 35.44 | 22.25 | 33.33 | 1200 | 325 | 189 | 93 | 933 |
| Nu_27 | 56.59 | 151.02 | 4.71 | 33.05 | 72.93 | 573 | 571 | 28 | 68 | 1491 |
| Nu_28 | 61.08 | 185.58 | 4.9 | 2.94 | 27.29 | 494 | 365 | 38 | 32 | 777 |
| Nu_29 | 39.89 | 215.07 | 16.02 | 7.43 | 27.17 | 246 | 289 | 20 | 12 | 418 |
| Nu_30 | 56.64 | 264.38 | 3.39 | 10.07 | 33.34 | 399 | 405 | 38 | 34 | 861 |
| Nu_31 | 16.38 | 26 | 1.34 | 1.28 | 9.31 | 221 | 115 | 15 | 6 | 257 |

Table S3: Behavior classes for coypu in duration and frequency per catch period in minutes.

|  | Variable | Mean | Standard deviation | Minimum | Maximum | N | Median |
| --- | --- | --- | --- | --- | --- | --- | --- |
| duration | movement | 38.76 | 23.95 | 9.31 | 103.93 | 31 | 33.33 |
|  | exploration | 144.58 | 90.85 | 0.12 | 292.32 | 31 | 162.59 |
|  | resting | 120.27 | 89.34 | 9.13 | 292.35 | 31 | 84.85 |
|  | comfort | 6.06 | 7.64 | 0 | 35.44 | 31 | 3.00 |
|  | foraging | 10.47 | 12.00 | 0 | 49.99 | 31 | 5.82 |
| frequency | movement | 940.10 | 643.80 | 52.00 | 2496.00 | 31 | 777.00 |
|  | exploration | 814.58 | 632.19 | 1.00 | 2185.00 | 31 | 561.00 |
|  | resting | 354.61 | 203.94 | 26.00 | 803.00 | 31 | 320.00 |
|  | comfort | 34.48 | 36.35 | 0 | 189.00 | 31 | 24.00 |
|  | foraging | 30.10 | 36.7 | 0 | 180.00 | 31 | 24.00 |

Table S4: Extracted behavioral patterns of the total data set with minimum, median, and maximum values of duration time and frequency, shown in minutes per catch period (maximum six hours) for coypu.

|  | Duration per catch period | | | Frequency per catch period | | |
| --- | --- | --- | --- | --- | --- | --- |
| Behavioral pattern | **Minimum** | **Median** | **Maximum** | **Minimum** | **Median** | **Maximum** |
| being startled | 0.00 | 0.00 | 1.33 | 0.00 | 0.00 | 14.00 |
| pausing | 0.00 | 0.00 | 1.04 | 0.00 | 0.00 | 11.00 |
| trembling | 0.00 | 0.07 | 16.52 | 0.00 | 2.00 | 105.00 |
| gnawing | 0.00 | 0.84 | 32.74 | 0.00 | 8.00 | 265 |
| nasalizing | 0.00 | 16.37 | 59.31 | 0.00 | 222.00 | 849 |
| grooming | 0.00 | 2.51 | 18.92 | 00.00 | 21.00 | 84.00 |
| coprophagia | 0.00 | 0.11 | 14.04 | 0.00 | 1.00 | 31.00 |
| sleeping | 0.00 | 0.00 | 191.98 | 0.00 | 0.00 | 40.00 |

Table S5: Individual data and external factors per animal, used as predictors.

| Animal.ID | Trap type | Age | Gender | Season | Daytime | Tout | Weight class | Trec | Ttrap | Weather conditions | Full body index | SC Category |
| --- | --- | --- | --- | --- | --- | --- | --- | --- | --- | --- | --- | --- |
| Nu_01 | WG | adult | female | fall | evening | T0 | heavy | medium |  | cloudy | GS1 | SC1 |
| Nu_02 | SM | juvenile | male | fall | night | T0 | medium | high |  | cloudy | GS2 | SC2 |
| Nu_03 | SM | adult | male | winter | evening | T0 | medium | low |  | rainy | GS2 | SC1 |
| Nu_04 | WG | adult | female | winter | night | T2 | heavy | high |  | dry | GS1 | SC1 |
| Nu_05 | SM | juvenile | male | winter | evening | T1 | light | low |  | rainy | GS0 | SC3 |
| Nu_06 | WG | juvenile | female | winter | evening | T0 | light | medium |  | dry | GS0 | SC3 |
| Nu_07 | SM | adult | male | autumn | after-noon | T2 | heavy |  | Tt2 | dry | GS1 | SC1 |
| Nu_08 | WG | juvenile | female | fall | midday | T2 | medium | high |  | rainy | GS2 | SC3 |
| Nu_09 | SM | juvenile | female | fall | after-noon | T2 | light | high | Tt2 | rainy | GS0 | NA |
| Nu_10 | SM | juvenile | female | fall | after-noon | T2 | light | high | Tt2 | cloudy | GS1 | SC3 |
| Nu_11 | WG | juvenile | female | fall | evening | T2 | medium | medium |  | cloudy | GS1 | SC2 |
| Nu_12 | SM | juvenile | female | fall | evening | T2 | light | medium | Tt2 | dry | GS1 | SC2 |
| Nu_13 | WG | adult | male | fall | after-noon | T1 | medium | high |  | foggy | GS2 | SC2 |
| Nu_14 | WG | adult | male | fall | night | T1 | heavy | high |  | foggy | GS0 | SC2 |
| Nu_15 | WG | adult | female | fall | night | T2 | medium | high |  | dry | GS2 | SC3 |
| Nu_16 | WG | adult | male | fall | night | T2 | medium | medium |  | rainy | GS0 | SC1 |
| Nu_17 | WG | adult | female | winter | evening | T1 | heavy | medium |  | rainy | GS1 | SC1 |
| Nu_18 | WG | juvenile | female | winter | evening |  | light | high |  | cloudy | GS0 | SC2 |
| Nu_19 | WG | adult | male | winter | evening | T2 | heavy | medium |  | dry | GS1 | SC1 |
| Nu_20 | SM | adult | male | winter | night | T2 | heavy | low | Tt2 | windy | GS0 | SC1 |
| Nu_21 | WB | adult | male | winter | morning | T1 | heavy | medium | Tt1 | dry | GS2 | SC1 |
| Nu_22 | WB | adult | male | winter | night | T0 | heavy | high | Tt1 | foggy | GS1 | SC2 |
| Nu_23 | WB | adult | female | winter | night | T1 | medium | high | Tt1 | dry | GS0 | SC3 |
| Nu_24 | SM | adult | female | winter | night | T1 | heavy | low | Tt1 | dry | GS1 | SC1 |
| Nu_25 | SM | adult | male | spring | night | T1 | heavy | low | Tt1 | dry | GS3 | SC1 |
| Nu_26 | WG | adult | male | spring | morning | T2 | medium | low |  | windy | GS2 | SC2 |
| Nu_27 | WG | adult | male | spring | night | T0 | medium | medium |  | dry | GS0 | SC3 |
| Nu_28 | WG | adult | male | spring | night | T0 | medium | medium |  | dry | GS0 | SC3 |
| Nu_29 | SM | adult | male | spring | evening | T1 | heavy | low | Tt2 | dry | GS1 | SC1 |
| Nu_30 | WG | juvenile | female | spring | evening | T1 | medium | high |  | dry | GS0 | SC2 |
| Nu_31 | WG | adult | female | spring | night | T1 | medium | high |  | dry | GS0 | SC2 |

### **Models**

#### External models

Table S6: Results of coypu **exploration behavior** during live trapping calculated using generalized linear models.
Behavior in duration time (A) and frequency of classes (B).

|  |  | (A) duration AIC 304.1 R^2^ 0.9631753 Observations 30 df 29 | | |  |  |
| --- | --- | --- | --- | --- | --- | --- |
| Type | **Variable** | **Estimate** | **SE** | ***p*** | **Sign.** | **n** |
|  | intercept | 18.361 | 41.276 | 0.6699 |  |  |
| External factors | WG trap | 74.757 | 72.591 | 0.3373 |  | 17 |
|  | WB trap | -181.782 | 104.264 | 0.1248 |  | 3 |
|  | spring | -43.507 | 52.271 | 0.4327 |  | 7 |
|  | winter | 23.802 | 49.723 | 0.6467 |  | 12 |
|  | morning | 93.376 | 68.135 | 0.2129 |  | 2 |
|  | midday | 72.693 | 69.443 | 0.3300 |  | 1 |
|  | evening | 31.542 | 37.748 | 0.4310 |  | 11 |
|  | night | 142.836 | 45.650 | 0.0166 | * | 13 |
|  | dry | -19.892 | 37.748 | 0.6145 |  | 15 |
|  | foggy | 61.933 | 70.748 | 0.4076 |  | 3 |
|  | rainy | 30.193 | 46.924 | 0.5404 |  | 6 |
|  | windy | -10.301 | 72.667 | 0.8913 |  | 2 |
|  | ToutT1 | 41.569 | 28.290 | 0.1852 |  | 11 |
|  | ToutT2 | 7.742 | 30.654 | 0.8079 |  | 12 |
|  | WG trap:spring | -160.651 | 56.136 | 0.0243 | * |  |
|  | WG trap:windy | 278.333 | 124.509 | 0.0605 | . |  |
|  | WG trap:dry | 75.765 | 62.313 | 0.2634 |  |  |
|  | WB trap:dry | 86.716 | 109.624 | 0.44549 |  |  |
|  | WG trap:rainy | -16.997 | 69.893 | 0.8148 |  |  |
|  | WG trap: evening | 32.259 | 70.445 | 0.6609 |  |  |
|  | WG trap:night | -54.696 | 68.135 | 0.4485 |  |  |

All variables not listed could not be calculated due to insufficient sample numbers.

|  |  | (B) frequency AIC 466.34 R^2^ 0.790621 Observations 31 df 30 | | |  |  |
| --- | --- | --- | --- | --- | --- | --- |
| Type | **Variable** | **Estimate** | **SE** | ***p*** | **Sign.** | **n** |
|  | intercept | 216.30 | 248.70 | 0.395891 |  |  |
| External factors | WG trap | 722.66 | 168.64 | 0.000446 | *** | 17 |
|  | WB trap | -577.66 | 377.63 | 0.143475 |  | 3 |
|  | spring | -261.95 | 239.54 | 0.288570 |  | 7 |
|  | winter | 678.19 | 220.69 | 0.006554 | ** | 12 |
|  | morning | 428.89 | 481.25 | 0.384574 |  | 2 |
|  | midday | 1009.39 | 477.54 | 0.048759 | * | 1 |
|  | evening | 127.77 | 276.80 | 0.649901 |  | 11 |
|  | night | 68.02 | 261.61 | 0.797813 |  | 13 |
|  | dry | -106.92 | 233.81 | 0.652934 |  | 15 |
|  | foggy | -172.60 | 311.96 | 0.586885 |  | 3 |
|  | rainy | 27.65 | 254.48 | 0.914670 |  | 6 |
|  | windy | -255.71 | 431.84 | 0.561125 |  | 2 |

Table S7: Results of coypu **movement behavior** during live trapping calculated using generalized linear models.
Behavior in duration time (A) and frequency of classes (B).

|  |  | (A) duration AIC 269.01 R^2^ 0.646862 Observations 30 df 29 | | |  |  |
| --- | --- | --- | --- | --- | --- | --- |
| Type | **Variable** | **Estimate** | **SE** | ***p*** | **Sign.** | **n** |
|  | intercept | 57.226 | 15.634 | 0.00232 | ** |  |
| External factors | WG trap | 19.683 | 8.904 | 0.04301 | * | 17 |
|  | WB trap | 6.133 | 19.094 | 0.75249 |  | 3 |
|  | spring | -6.171 | 16.127 | 0.70735 |  | 7 |
|  | winter | 1.993 | 13.834 | 0.88734 |  | 12 |
|  | morning | -15.050 | 24.869 | 0.55411 |  | 2 |
|  | midday | 42.106 | 23.968 | 0.09935 | . | 1 |
|  | evening | 3.400 | 14.398 | 0.81652 |  | 11 |
|  | night | -12.731 | 13.735 | 0.36865 |  | 13 |
|  | dry | -15.250 | 15.012 | 0.32581 |  | 15 |
|  | foggy | -19.462 | 17.721 | 0.28942 |  | 3 |
|  | rainy | -10.860 | 15.122 | 0.48368 |  | 6 |
|  | windy | -4.473 | 28.236 | 0.87623 |  | 2 |
|  | ToutT1 | -16.832 | 9.880 | 0.10908 |  | 11 |
|  | ToutT2 | -20.215 | 13.229 | 0.14729 |  | 12 |

|  |  | (B) frequency AIC 473.72 R^2^ 0.6683225 Observations 31 df 30 | | |  |  |
| --- | --- | --- | --- | --- | --- | --- |
| Type | **Variable** | **Estimate** | **SE** | ***p*** | **Sign.** | **n** |
|  | intercept | 296.3 | 221.1 | 0.19404 |  |  |
| External factors | WG trap | 646.1 | 180.2 | 0.00165 | ** | 17 |
|  | WB trap | -160.2 | 349.5 | 0.65111 |  | 3 |
|  | spring | -295.6 | 237.9 | 0.22702 |  | 7 |
|  | winter | 191.9 | 224.9 | 0.40275 |  | 11 |
|  | morning | 168.7 | 460.8 | 0.71776 |  | 2 |
|  | midday | 1553.7 | 502.6 | 0.00533 | ** | 1 |
|  | evening | 497.2 | 305.2 | 0.11758 |  | 11 |
|  | night | 143.3 | 295.0 | 0.63197 |  | 13 |

Table S8: Results of coypu **resting behavior** during live trapping calculated using generalized linear models.
Behavior in duration time (A) and frequency of classes (B).

|  |  | (A) duration AIC 320.1 R^2^ 0.941587 Observations 30 df 29 | | |  |  |
| --- | --- | --- | --- | --- | --- | --- |
| Type | **Variable** | **Estimate** | **SE** | ***p*** | **Sign.** | **n** |
|  | intercept | 208.147 | 113.633 | 0.1265 |  |  |
| External factors | WG trap | -311.945 | 152.233 | 0.0957 | . | 17 |
|  | WB trap | 48.443 | 155.388 | 0.7678 |  | 3 |
|  | spring | -137.113 | 124.836 | 0.3221 |  | 7 |
|  | winter | -221.290 | 109.645 | 0.0996 | . | 12 |
|  | morning | 2.323 | 124.836 | 0.9859 |  | 2 |
|  | midday | -58.508 | 100.665 | 0.5863 |  | 1 |
|  | evening | -29.463 | 69.985 | 0.6912 |  | 11 |
|  | night | -51.817 | 101.198 | 0.6304 |  | 13 |
|  | dry | 192.353 | 69.985 | 0.0404 | * | 15 |
|  | foggy | 248.617 | 110.106 | 0.0735 | . | 3 |
|  | rainy | 127.457 | 69.985 | 0.1282 |  | 6 |
|  | windy | 234.007 | 202.395 | 0.2998 |  | 2 |
|  | ToutT1 | -16.227 | 69.958 | 0.8258 |  | 11 |
|  | ToutT2 | -110.777 | 124.836 | 0.4155 |  | 12 |
|  | WG trap: evening | 174.127 | 112.574 | 0.1826 |  |  |
|  | WG trap: morning | -320.667 | 217.624 | 0.2006 |  |  |
|  | WG trap:night | 57.747 | 124.836 | 0.6631 |  |  |
|  | WG trap:spring | 299.758 | 148.558 | 0.0997 | . |  |
|  | WG trap:winter | 133.489 | 125.180 | 0.3350 |  |  |
|  | WG trap:dry | -82.404 | 103.715 | 0.4629 |  |  |
|  | WB trap:dry | 44.990 | 162.393 | 0.7928 |  |  |
|  | WG trap:rainy | 69.210 | 103.715 | 0.5341 |  |  |
|  | WG trap:ToutT1 | -89.102 | 86.258 | 0.3490 |  |  |
|  | WG trap: ToutT2 | 145.397 | 133.536 | 0.3259 |  |  |

|  |  | (B) frequency AIC 404.31 R^2^ 0.6478564 Observations 31 df 30 | | |  |  |
| --- | --- | --- | --- | --- | --- | --- |
| Type | **Variable** | **Estimate** | **SE** | ***p*** | **Sign.** | **n** |
|  | intercept | 116.233 | 72.174 | 0.12155 |  |  |
| External factors | WG trap | 175.067 | 58.825 | 0.00697 | ** | 17 |
|  | WB trap | -15.862 | 114.071 | 0.89067 |  | 3 |
|  | spring | 54.350 | 77.642 | 0.49126 |  | 7 |
|  | winter | 195.934 | 73.413 | 0.01402 | * | 12 |
|  | morning | -68.478 | 150.394 | 0.65334 |  | 2 |
|  | midday | 511.699 | 164.044 | 0.00499 | ** | 1 |
|  | evening | 133.969 | 99.638 | 0.26499 |  | 11 |
|  | night | 7.781 | 96.295 | 0.93633 |  | 13 |

Table S9: Results of coypu **comfort behavior** during live trapping calculated using generalized linear models.
Behavior in duration time (A) and frequency of classes (B).

|  |  | (A) duration AIC 181.73 R^2^ 0.9131026 Observations 31 df 29 | | |  |  |
| --- | --- | --- | --- | --- | --- | --- |
| Type | **Variable** | **Estimate** | **SE** | ***p*** | **Sign.** | **n** |
|  | intercept | 4.8730 | 9.3647 | 0.6189 |  |  |
| External factors | WG trap | -12.1674 | 12.7409 | 0.3714 |  | 17 |
|  | WB trap | -6.4869 | 11.3806 | 0.5865 |  | 3 |
|  | spring | -7.1109 | 10.3988 | 0.5161 |  | 7 |
|  | winter | -7.8053 | 9.1163 | 0.4202 |  | 12 |
|  | morning | -1.8330 | 10.4571 | 0.8658 |  | 2 |
|  | midday | -3.1926 | 8.8072 | 0.7277 |  | 1 |
|  | evening | 0.6426 | 5.8535 | 0.9157 |  | 11 |
|  | night | -2.5830 | 8.1269 | 0.7599 |  | 13 |
|  | dry | 2.7209 | 4.6397 | 0.5760 |  | 15 |
|  | foggy | 15.0022 | 8.7739 | 0.1310 |  | 3 |
|  | rainy | 5.1397 | 4.6397 | 0.3046 |  | 6 |
|  | windy | 14.2718 | 16.1035 | 0.4049 |  | 2 |
|  | ToutT1 | 14.9322 | 6.2238 | 0.0475 | * | 11 |
|  | ToutT2 | -6.4765 | 9.8292 | 0.5310 |  | 12 |
|  | WG trap: evening | 5.9949 | 9.8257 | 0.5611 |  |  |
|  | WG trap: morning | 14.2702 | 15.4666 | 0.3869 |  |  |
|  | WG trap:night | 2.1430 | 10.4571 | 0.8435 |  |  |
|  | WG trap:spring | 16.3189 | 11.0460 | 0.1831 |  |  |
|  | WG trap:winter | 13.4492 | 9.0673 | 0.1816 |  |  |
|  | WB trap:ToutT1 | -2.7209 | 12.2871 | 0.8311 |  |  |
|  | WG trap:ToutT1 | -19.6899 | 7.6871 | 0.0375 | * |  |
|  | WG trap: ToutT2 | 13.2939 | 10.1854 | 0.2331 |  |  |

|  |  | (B) frequency AIC 308.48 R^2^ 0.6109006 Observations 31 df 30 | | |  |  |
| --- | --- | --- | --- | --- | --- | --- |
| Type | **Variable** | **Estimate** | **SE** | ***p*** | **Sign.** | **n** |
|  | intercept | 1.343 | 19.496 | 0.9458 |  |  |
| External factors | WG trap | 8.810 | 13.220 | 0.5136 |  | 17 |
|  | WB trap | -20.094 | 29.603 | 0.5059 |  | 3 |
|  | spring | 21.588 | 18.778 | 0.2653 |  | 7 |
|  | winter | 11.292 | 17.301 | 0.5222 |  | 12 |
|  | morning | 81.994 | 37.726 | 0.0433 | * | 2 |
|  | midday | -19.398 | 37.435 | 0.6107 |  | 1 |
|  | evening | 16.402 | 21.699 | 0.4595 |  | 11 |
|  | night | 10.358 | 20.508 | 0.6196 |  | 13 |

### **Paired catches**

Table S10: Behavior classes for each individual (coypu, paired catch) in duration and frequency per catch period.

|  | Duration [minutes] | | | | | | Frequency [number of events] | | | | | |
| --- | --- | --- | --- | --- | --- | --- | --- | --- | --- | --- | --- | --- |
| Animal ID | exploration | resting | comfort | foraging | movement | interaction | exploration | resting | comfort | foraging | movement | interaction |
| Nu_ DF1 | 68.06 | 104.73 | 2.65 | 2.36 | 25.64 | 104.73 | 207 | 202 | 8 | 6 | 294 | 120 |
| Nu_ DF2 | 30.82 | 150.41 | 0.45 | 8.59 | 24.57 | 150.41 | 138 | 213 | 4 | 13 | 283 | 152 |
| Nu_ DF3 | 26.13 | 248.59 | 0.72 | 32.01 | 52.15 | 248.59 | 104 | 360 | 8 | 72 | 462 | 267 |
| Nu_ DF4 | 17.99 | 263.94 | 1.13 | 21.13 | 51.16 | 263.94 | 110 | 320 | 7 | 55 | 455 | 254 |
| Nu_ DF5 | 12.80 | 37.15 | 9.42 | 0.76 | 57.36 | 37.15 | 61 | 415 | 25 | 5 | 591 | 345 |
| Nu_ DF6 | 10.56 | 280.78 | 4.80 | 2.63 | 60.77 | 280.78 | 57 | 423 | 34 | 9 | 565 | 346 |

Table 19: Classes of the paired catch dataset with minimum, median, and maximum values of duration time (d) and frequency (f), shown in minutes per catch period.

| Behavior class | | Minimum | Median | Mean | Maximum | Std.dev. | N |
| --- | --- | --- | --- | --- | --- | --- | --- |
| duration | **interaction** | 76.55 | 201.68 | 183.41 | 253.98 | 70.47 | 6 |
|  | **movement** | 24.57 | 51.65 | 45.26 | 60.77 | 16.01 | 6 |
|  | **exploration** | 10.56 | 22.06 | 27.73 | 68.06 | 21.21 | 6 |
|  | **resting** | 37.15 | 199.50 | 180.93 | 280.78 | 98.84 | 6 |
|  | **comfort** | 0.45 | 1.89 | 3.20 | 9.42 | 3.45 | 6 |
|  | **foraging** | 0.76 | 5.61 | 11.25 | 32.01 | 12.64 | 6 |
| frequency | **interaction** | 120.00 | 260.50 | 247.33 | 346.00 | 94,87 | 6 |
|  | **movement** | 283.00 | 458.50 | 441.83 | 591.00 | 130.22 | 6 |
|  | **exploration** | 57.00 | 107.00 | 112.83 | 207.00 | 55.48 | 6 |
|  | **resting** | 202.00 | 340.00 | 322.17 | 423.00 | 96.52 | 6 |
|  | **comfort** | 4.00 | 8.00 | 14.33 | 34.00 | 12.17 | 6 |
|  | **foraging** | 5.00 | 11.00 | 26.67 | 72.00 | 29.16 | 6 |

Table S11: Individual and external factors per animal (coypu, paired catched).

| AnimalID | Trap type | Age | Gender | Season | Daytime | Tout | Weight class | Trec | Ttrap | Weather conditions | Full body index | SC_Category |
| --- | --- | --- | --- | --- | --- | --- | --- | --- | --- | --- | --- | --- |
| Nu_ DF1 | SM | juvenile | female | winter | night | T1 | light | low | T0 | sleet | GS3 | SC2 |
| Nu_ DF2 | SM | juvenile | male | winter | night | T1 | light | medium | T0 | sleet | GS0 | SC2 |
| Nu_ DF3 | SM | juvenile | female | winter | evening | T1 | light | medium | T1 | rainy | GS0 | SC2 |
| Nu_ DF4 | SM | juvenile | female | winter | evening | T1 | light | low | T1 | rainy | GS0 | SC1 |
| Nu_ DF5 | SM | juvenile | female | winter | morning | T1 | light | low | T2 | dry | GS0 | SC1 |
| Nu_ DF6 | SM | juvenile | female | winter | morning | T1 | light | medium | T2 | dry | GS0 | SC1 |

### Occurrence of animals outside the trap

Table S12: Overview of the duration [minutes] and frequency of presence of animals captured with photo traps at the trapping site, listed by trap type.

| Trap type | N | Sighting of coypu | Sighting of animal | Duration per trap type | Duration per catch period | Min | Median | Mean | Max |
| --- | --- | --- | --- | --- | --- | --- | --- | --- | --- |
| wire grid | 5 | 17 | 26 | 73.97 | 2.84 | 0.88 | 11.95 | 14.79 | 34.90 |
| sheet metal | 4 | 7 | 9 | 90.95 | 10.11 | 3.63 | 19.26 | 22.74 | 48.80 |
| wooden box | 3 | 0 | 7 | 157.00 | 22.43 | 43.98 | 51.03 | 52.33 | 61.98 |
| Total | 12 | 24 | 42 | 321.92 | 7.66 |  |  |  |  |

## **Raccoon**

### Behavioral data

Table S13: Behavior classes for each individual (raccoon) in duration and frequency per catch period.

|  | Duration [minutes] | | | | | Frequency [number of events] | | | | |
| --- | --- | --- | --- | --- | --- | --- | --- | --- | --- | --- |
| Animal ID | exploration | resting | comfort | foraging | movement | exploration | resting | comfort | foraging | movement |
| Wb_01 | 179.28 | 3.65 | 8.04 | 0.58 | 5.99 | 557 | 68 | 24 | 3 | 105 |
| Wb_02 | 282.73 | 53.04 | 9.68 | 0 | 11.64 | 935 | 234 | 36 | 0 | 270 |
| Wb_03 | 222.51 | 78.96 | 3.48 | 6.73 | 49.7 | 1250 | 421 | 41 | 11 | 753 |
| Wb_04 | 101.97 | 186.49 | 44.9 | 1.55 | 11.77 | 243 | 205 | 65 | 2 | 65 |
| Wb_05 | 234.51 | 94.38 | 21.56 | 0 | 11.64 | 1069 | 281 | 32 | 0 | 271 |
| Wb_06 | 174.78 | 146.82 | 2.27 | 8.65 | 6.58 | 816 | 299 | 25 | 30 | 187 |
| Wb_07 | 96.31 | 207.17 | 46.01 | 0.09 | 8.35 | 409 | 209 | 66 | 2 | 198 |
| Wb_08 | 85.17 | 188.74 | 56.33 | 0.07 | 4.69 | 217 | 98 | 90 | 1 | 111 |

Table S14: Behavior classes for raccoon in duration and frequency per catch period in minutes.

|  | Variable | Mean | Standard deviation | Minimum | Maximum | N | Median |
| --- | --- | --- | --- | --- | --- | --- | --- |
| duration | movement | 13.79 | 14.77 | 4.69 | 49.70 | 8 | 9.99 |
|  | exploration | 172.16 | 72.66 | 85.17 | 282.73 | 8 | 177.03 |
|  | resting | 119.91 | 73.51 | 3.65 | 207.17 | 8 | 120.60 |
|  | comfort | 24.03 | 21.80 | 2.27 | 56.33 | 8 | 15.62 |
|  | foraging | 2.21 | 3.46 | 0 | 8.65 | 8 | 0.34 |
| frequency | movement | 245.00 | 218.73 | 65.00 | 753.00 | 8 | 192.50 |
|  | exploration | 687.00 | 387.90 | 217.00 | 1250.00 | 8 | 686.50 |
|  | resting | 226.88 | 112.45 | 68.00 | 421.00 | 8 | 221.50 |
|  | comfort | 47.38 | 23.69 | 24.00 | 90.00 | 8 | 38.50 |
|  | foraging | 6.13 | 10.27 | 0 | 30.00 | 8 | 2.00 |

Table S15: Individual data and external factors per animal (raccoon), used as predictors.

| Animal.ID | Trap type | Age | Gender | Season | Daytime | Tout | Weight class | Trec | Ttrap | Weather conditions | Full body index | SC_Category |
| --- | --- | --- | --- | --- | --- | --- | --- | --- | --- | --- | --- | --- |
| Wb _01 | WG | juvenile | male | fall | night | T1 | light |  |  | windy | GS0 | SC2 |
| Wb _02 | WG | juvenile | male | fall | night | T1 | light |  |  | humid | GS1 |  |
| Wb _03 | WG | juvenile | female | fall | evening | T1 | light |  |  | cloudy | GS1 | SC2 |
| Wb _04 | WB | juvenile | male | fall | night | T1 | medium |  |  | cloudy | GS2 | SC1 |
| Wb _05 | WG | juvenile | male | fall | night | T1 | medium | medium |  | sunny | GS1 |  |
| Wb _06 | WG | juvenile | male | fall | evening | T1 | heavy | low |  | dry | GS3 | SC2 |
| Wb _07 | WB | juvenile | male | fall | night | T0 | light | low | Tt2 | rainy | GS2 |  |
| Wb _08 | WB | adult | male | winter | night | T0 | heavy | low |  | dry | GS4 | SC2 |

### Models

#### Individual models

Table S16: Results of raccoon **movement behavior** during live trapping calculated using generalized linear models.
Behavior in duration time (A) and frequency of classes (B).

|  |  | (A) duration AIC 39.146 R^2^ 0.988286 Observations 8 df 7 | | |  |  |
| --- | --- | --- | --- | --- | --- | --- |
| Type | **Variable** | **Estimate** | **SE** | ***p*** | **Sign.** | **n** |
|  | intercept | 46.675 | 2.864 | 8.30e-05 | *** |  |
| Individual data | male | -41.040 | 2.443 | 7.36e-05 | *** | 7 |
|  | weight class light | 3.025 | 1.931 | 0.1923 |  | 4 |
|  | weight class medium | 6.070 | 2.115 | 0.0455 | * | 2 |

|  |  | (B) frequency AIC 97.9 R^2^ 0.8938132 Observations 8 df 7 | | |  |  |
| --- | --- | --- | --- | --- | --- | --- |
| Type | **Variable** | **Estimate** | **SE** | ***p*** | **Sign.** | **n** |
|  | intercept | 681.33 | 124.14 | 0.00274 | ** |  |
| Individual data | male | -570.33 | 91.09 | 0.00152 | ** | 7 |
|  | juvenile | 71.67 | 91.09 | 0.46706 |  | 7 |

Table S17: Results of raccoon **resting behavior** during live trapping calculated using generalized linear models.
Behavior in frequency of classes (B).

|  |  | (B) frequency AIC 96.846 R^2^ 0.7863787 Observations 8 df 7 | | |  |  |
| --- | --- | --- | --- | --- | --- | --- |
| Type | **Variable** | **Estimate** | **SE** | ***p*** | **Sign.** | **n** |
|  | intercept | 348.67 | 121.27 | 0.0638 | . |  |
| Individual data | male | -250.67 | 91.67 | 0.0717 | . | 7 |
|  | juvenile | 201.00 | 112.28 | 0.1713 |  | 7 |
|  | weight class light | -128.67 | 91.67 | 0.2551 |  | 4 |
|  | weight class medium | -56.00 | 97.23 | 0.6050 |  | 2 |

Table S18: Results of raccoon **foraging behavior** during live trapping calculated using generalized linear models.

Behavior in duration time (A) and frequency of classes (B).

|  |  | (A) duration AIC 20.737 R^2^ 0.9833476 Observations 8 df 7 | | |  |  |
| --- | --- | --- | --- | --- | --- | --- |
| Type | **Variable** | **Estimate** | **SE** | ***p*** | **Sign.** | **n** |
|  | intercept | 6.5767 | 1.0421 | 0.00804 | ** |  |
| Individual data | male | -6.5067 | 0.7877 | 0.00372 | ** | 7 |
|  | weight class light | -8.4267 | 0.7877 | 0.00175 | ** | 4 |
|  | weight class medium | -7.8750 | 0.8355 | 0.002533 | ** | 2 |
|  | juvenile | 8.5800 | 0.9648 | 0.00300 | ** | 7 |

|  |  | (B) frequency AIC 33.244 R^2^ 0.9909773 Observations 8 df 7 | | |  |  |
| --- | --- | --- | --- | --- | --- | --- |
| Type | **Variable** | **Estimate** | **SE** | ***p*** | **Sign.** | **n** |
|  | intercept | 10.333 | 2.277 | 0.020033 | * |  |
| Individual data | male | -9.333 | 1.721 | 0.012308 | * | 7 |
|  | juvenile | 29..000 | 2.108 | 0.000831 | *** | 7 |
|  | weight class light | -28.333 | 1.721 | 0.000488 | *** | 4 |
|  | weight class medium | -29.000 | 2.108 | 0.000543 | *** | 2 |

Table S19: Results of raccoon **comfort behavior** during live trapping calculated using generalized linear models.

Behavior in duration time (A) and frequency of classes (B).

|  |  | (A) duration AIC 74.747 R^2^ 0.641012 Observations 8 df 7 | | |  |  |
| --- | --- | --- | --- | --- | --- | --- |
| Type | **Variable** | **Estimate** | **SE** | ***p*** | **Sign.** | **n** |
|  | intercept | 38.57 | 30.47 | 0.295 |  |  |
| Individual data | male | 17.76 | 23.03 | 0.497 |  | 7 |
|  | juvenile | -54.06 | 28.21 | 0.151 |  | 7 |
|  | weight class light | 18.97 | 23.03 | 0.470 |  | 4 |
|  | weight class medium | 30.96 | 24.43 | 0.295 |  | 2 |

|  |  | (B) frequency AIC 74.473 R^2^ 0.6228877 Observations 8 df 7 | | |  |  |
| --- | --- | --- | --- | --- | --- | --- |
| Type | **Variable** | **Estimate** | **SE** | ***p*** | **Sign.** | **n** |
|  | intercept | 90.00 | 19.24 | 0.00947 | ** |  |
| Individual data | juvenile | -65.00 | 27.21 | 0.07530 | . | 7 |
|  | weight class light | 16.75 | 21.51 | 0.47974 |  | 4 |
|  | weight class medium | 23.50 | 23.57 | 0.037515 |  | 2 |

#### External models

Table S20: Results of raccoon **exploration behavior** during live trapping calculated using generalized linear models.
Behavior in duration time (A) and frequency of classes (B).

|  |  | (A) duration AIC 65.885 R^2^ 0.9935278 Observations 8 df 7 | | |  |  |
| --- | --- | --- | --- | --- | --- | --- |
| Type | **Variable** | **Estimate** | **SE** | ***p*** | **Sign.** | **n** |
|  | intercept | 214.78 | 13.39 | 0.0396 | * |  |
| External factors | WB trap | -69.58 | 20.46 | 0.1821 |  | 3 |
|  | night | -35.50 | 20.46 | 0.3328 |  | 6 |
|  | dry | -32.26 | 15.46 | 0.2845 |  | 2 |
|  | humid | 103.45 | 21.87 | 0.1326 |  | 1 |
|  | rainy | -13.39 | 20.46 | 0.6310 |  | 1 |
|  | sunny | 55.23 | 21.87 | 0.2400 |  | 1 |

All variables not listed could not be calculated due to insufficient sample numbers.

|  |  | (B) frequency AIC 107.16 R^2^ 0.9604883 Observations 8 df 7 | | |  |  |
| --- | --- | --- | --- | --- | --- | --- |
| Type | **Variable** | **Estimate** | **SE** | ***p*** | **Sign.** | **n** |
|  | intercept | 1148.0 | 176.7 | 0.0972 | . |  |
| External factors | WB trap | -803.0 | 204.0 | 0.1584 | *** | 3 |
|  | dry | -230.0 | 204.0 | 0.4619 |  | 2 |
|  | humid | -213.0 | 269.9 | 0.5746 |  | 1 |
|  | rainy | 64.0 | 269.9 | 0.8518 |  | 1 |
|  | sunny | -97.0 | 269.9 | 0.8187 |  | 1 |
|  | windy | -591.0 | 269.9 | 0.2727 |  | 1 |

Table S21: Results of raccoon **movement behavior** during live trapping calculated using generalized linear models.
Behavior in duration time (A) and frequency of classes (B).

|  |  | (A) duration AIC 68.331 R^2^ 0.7874979 Observations 8 df 7 | | |  |  |
| --- | --- | --- | --- | --- | --- | --- |
| Type | **Variable** | **Estimate** | **SE** | ***p*** | **Sign.** | **n** |
|  | intercept | 40.69 | 15.61 | 0.233 |  |  |
| External factors | WB trap | 14.79 | 23.84 | 0.646 |  | 3 |
|  | night | -34.70 | 23.84 | 0.383 |  | 6 |
|  | dry | -25.10 | 18.02 | 0.396 |  | 2 |
|  | humid | 5.65 | 25.48 | 0.861 |  | 1 |
|  | rainy | -12.43 | 23.84 | 0.694 |  | 1 |
|  | sunny | 5.65 | 25.48 | 0.861 |  | 1 |

|  |  | (B) frequency AIC 113.64 R^2^ 0.7204011 Observations 8 df 7 | | |  |  |
| --- | --- | --- | --- | --- | --- | --- |
| Type | **Variable** | **Estimate** | **SE** | ***p*** | **Sign.** | **n** |
|  | intercept | 600.0 | 265.0 | 0.265 |  |  |
| External factors | WB trap | 113.0 | 404.8 | 0.827 |  | 3 |
|  | night | -495.0 | 404.8 | 0.436 |  | 6 |
|  | dry | -260.0 | 306.0 | 0.552 |  | 2 |
|  | humid | 165.0 | 432.8 | 0.768 |  | 1 |
|  | rainy | -20.0 | 404.8 | 0.969 |  | 1 |
|  | sunny | 166.0 | 432.8 | 0.767 |  | 1 |
|  | windy | NA | NA | NA |  | 1 |

Table S22: Results of raccoon **resting behavior** during live trapping calculated using generalized linear models.
Behavior in duration time (A) and frequency of classes (B).

|  |  | (A) duration AIC 77.917 R^2^ 0.9715492 Observations 8 df 7 | | |  |  |
| --- | --- | --- | --- | --- | --- | --- |
| Type | **Variable** | **Estimate** | **SE** | ***p*** | **Sign.** | **n** |
|  | intercept | 95.3625 | 28.4100 | 0.184 |  |  |
| External factors | WB trap | 74.7250 | 32.8050 | 0.263 |  | 3 |
|  | dry | 35.0550 | 32.8050 | 0.479 |  | 2 |
|  | humid | -42.3225 | 43.3969 | 0.508 |  | 1 |
|  | rainy | 37.0825 | 43.3969 | 0.550 |  | 1 |
|  | sunny | -0.9852 | 43.3969 | 0.986 |  | 1 |
|  | windy | -91.7125 | 43.3969 | 0.281 |  | 1 |

|  |  | (B) frequency AIC 54.306 R^2^ 0.9993645 Observations 8 df 7 | | |  |  |
| --- | --- | --- | --- | --- | --- | --- |
| Type | **Variable** | **Estimate** | **SE** | ***p*** | **Sign.** | **n** |
|  | intercept | 417.250 | 6.495 | 0.00991 | ** |  |
| External factors | WB trap | 140.750 | 9.922 | 0.04480 | * | 3 |
|  | night | -349.250 | 9.922 | 0.01808 | * | 6 |
|  | dry | -114.500 | 7.500 | 0.04164 | * | 2 |
|  | humid | 166.000 | 10.607 | 0.04062 | * | 1 |
|  | rainy | 0.250 | 9.922 | 0.98396 |  | 1 |
|  | sunny | 213.000 | 10.607 | 0.03168 | * | 1 |

Table S23: Results of raccoon **comfort behavior** during live trapping calculated using generalized linear models.
Behavior in duration time (A) and frequency of classes (B).

|  |  | (A) duration AIC 53.687 R^2^ 0.966856 Observations 8 df 7 | | |  |  |
| --- | --- | --- | --- | --- | --- | --- |
| Type | **Variable** | **Estimate** | **SE** | ***p*** | **Sign.** | **n** |
|  | intercept | 2.875 | 3.712 | 0.48184 |  |  |
| External factors | WB trap | 32.362 | 4.792 | 0.00251 | ** | 3 |
|  | winter | 10.875 | 6.429 | 0.16599 |  | 1 |
|  | night | 10.218 | 4.792 | 0.09994 | . | 6 |

|  |  | (B) frequency AIC 65.833 R^2^ 0.9466124 Observations 8 df 7 | | |  |  |
| --- | --- | --- | --- | --- | --- | --- |
| Type | **Variable** | **Estimate** | **SE** | ***p*** | **Sign.** | **n** |
|  | intercept | 31.600 | 2.896 | 0.000112 | *** |  |
| External factors | WB trap | 33.900 | 5.418 | 0.001529 | ** | 3 |
|  | winter | 24.500 | 7.932 | 0.027193 | * | 1 |

Table S24: Results of raccoon **foraging behavior** during live trapping calculated using generalized linear models.
Behavior in duration time (A) and frequency of classes (B).

|  |  | (A) duration AIC 21.897 R^2^ 0.9753376 Observations 8 df 7 | | |  |  |
| --- | --- | --- | --- | --- | --- | --- |
| Type | **Variable** | **Estimate** | **SE** | ***p*** | **Sign.** | **n** |
|  | intercept | 6.2200 | 1.0168 | 0.003616 | *** |  |
| External factors | WB trap | 1.3567 | 0.8302 | 0.177565 |  | 3 |
|  | night | -7.4967 | 0.6563 | 0.000335 | *** | 6 |
|  | ToutT1 | 1.4700 | 0.8806 | 0.170360 |  | 6 |

|  |  | (B) frequency AIC 55.923 R^2^ 0.746687 Observations 8 df 7 | | |  |  |
| --- | --- | --- | --- | --- | --- | --- |
| Type | **Variable** | **Estimate** | **SE** | ***p*** | **Sign.** | **n** |
|  | intercept | 20.5000 | 4.3263 | 0.00516 | ** |  |
| External factors | WB trap | 0.6667 | 4.9956 | 0.89904 |  | 3 |
|  | night | -19.5000 | 5.5852 | 0.01744 | * | 6 |

## Endocrinological data

Table S25: Cortisol and DHEA values in serum and hair for coypu and raccoon.

|  | Species | Min | Median | Max | Mean | SD | n | Wilcoxon p |
| --- | --- | --- | --- | --- | --- | --- | --- | --- |
| Serum cortisol | coypu | 40.8 | 505.39 | 1313.52 | 531.27 | 274.65 | 56 | 3.45E-06 |
|  | raccoon | 49.37 | 140.22 | 283.23 | 145.65 | 70.92 | 23 |  |
| Hair cortisol | coypu | 28.14 | 88.80 | 207.66 | 93.07 | 45.77 | 58 | 4.93E-06 |
|  | raccoon | 5.54 | 17.66 | 51.44 | 20.74 | 11.35 | 16 |  |
| Serum DHEA | coypu | 0.18 | 0.35 | 1.7 | 0.40 | 0.23 | 56 | 4.86E-05 |
|  | raccoon | 0.30 | 1.12 | 24.58 | 4.43 | 7.15 | 23 |  |
| Hair DHEA | coypu | 4.58 | 44.23 | 427.99 | 72.82 | 74.15 | 55 | 0.8311 |
|  | raccoon | 20.53 | 51.68 | 448.77 | 75.24 | 101.67 | 16 |  |
| Quotient serum cortisol / hair cortisol | coypu | 0.53 | 6.02 | 21.03 | 7.11 | 5.19 | 56 | 0.4048 |
|  | raccoon | 2.38 | 5.60 | 39.84 | 9.45 | 9.37 | 16 |  |
| Quotient serum DHEA / hair DHEA | coypu | 0.001 | 0.007 | 0.087 | 0.013 | 0.017 | 56 | 0.0006963 |
|  | raccoon | 0.002 | 0.019 | 0.364 | 0.077 | 0.12 | 16 |  |
| Quotient serum cortisol / serum DHEA | coypu | 0.00134018 | 0.0074118 | 0.08733624 | 0.01320201 | 0.01717938 | 53 | 2.48E-08 |
|  | raccoon | 0.00249571 | 0.01944278 | 0.36434486 | 0.07666776 | 0.11912325 | 16 |  |

Table S26: Pearson-correlation for hormone derivates measured in serum and hair.

|  | SDHEA | SCORTISOl | HCORTISOL | HDHEA |
| --- | --- | --- | --- | --- |
| SDHEA | 1 | -0.2205505 | -0.2801297 | -0.01267164 |
| SCORTISOl | -0.2205505 | 1 | 0.3211241 | 0.19101566 |
| HCORTISOL | -0.2801297 | 0.3211241 | 1 | 0.15545060 |
| HDHEA | -0.01267164 | 0.19101566 | 0.15545060 | 1 |
